# Characterization of Hairpin Loops and Cruciforms Across 118,019 Genomes Spanning the Tree of Life

**DOI:** 10.1093/gbe/evag089

**Published:** 2026-04-04

**Authors:** Nikol Chantzi, Camille Moeckel, Candace S Y Chan, Akshatha Nayak, Guliang Wang, Ioannis Mouratidis, Dionysios Chartoumpekis, Karen M Vasquez, Ilias Georgakopoulos-Soares

**Affiliations:** Institute for Personalized Medicine, Department of Molecular and Precision Medicine, The Pennsylvania State University College of Medicine, Hershey, PA, USA; Division of Pharmacology and Toxicology, College of Pharmacy, The University of Texas at Austin, Dell Pediatric Research Institute, Austin, TX, USA; Institute for Personalized Medicine, Department of Molecular and Precision Medicine, The Pennsylvania State University College of Medicine, Hershey, PA, USA; Institute for Personalized Medicine, Department of Molecular and Precision Medicine, The Pennsylvania State University College of Medicine, Hershey, PA, USA; Division of Pharmacology and Toxicology, College of Pharmacy, The University of Texas at Austin, Dell Pediatric Research Institute, Austin, TX, USA; Institute for Personalized Medicine, Department of Molecular and Precision Medicine, The Pennsylvania State University College of Medicine, Hershey, PA, USA; Division of Pharmacology and Toxicology, College of Pharmacy, The University of Texas at Austin, Dell Pediatric Research Institute, Austin, TX, USA; Division of Pharmacology and Toxicology, College of Pharmacy, The University of Texas at Austin, Dell Pediatric Research Institute, Austin, TX, USA; Institute for Personalized Medicine, Department of Molecular and Precision Medicine, The Pennsylvania State University College of Medicine, Hershey, PA, USA; Division of Pharmacology and Toxicology, College of Pharmacy, The University of Texas at Austin, Dell Pediatric Research Institute, Austin, TX, USA; Department of Internal Medicine, Division of Endocrinology, Medical School, University of Patras, Patras, Greece; Division of Pharmacology and Toxicology, College of Pharmacy, The University of Texas at Austin, Dell Pediatric Research Institute, Austin, TX, USA; Institute for Personalized Medicine, Department of Molecular and Precision Medicine, The Pennsylvania State University College of Medicine, Hershey, PA, USA; Division of Pharmacology and Toxicology, College of Pharmacy, The University of Texas at Austin, Dell Pediatric Research Institute, Austin, TX, USA

**Keywords:** evolution, inverted repeats, hairpins, cruciforms, genomes

## Abstract

Inverted repeats (IRs) can form alternative DNA secondary structures, including hairpins and cruciforms, which have a multitude of functional roles and have been associated with genomic instability. However, their prevalence across diverse organismal genomes remains only partially understood. Here, we examine the prevalence of perfect IRs, which do not have mismatches in their arms, across 118,019 complete organismal genomes. Our comprehensive analysis across taxonomic subdivisions reveals significant differences in the distribution, frequency, and biophysical properties of perfect IRs among these genomes. We identify a total of 33,558,920 perfect IRs and show a highly variable density across different organisms, with strikingly distinct patterns observed in Viruses, Bacteria, Archaea, and Eukaryota. We report IRs with perfect arms of extreme lengths, which can extend to hundreds of thousands of base pairs. Our findings reveal that Bacteria possess the highest IR density. Additionally, this study reveals the enrichment of IRs at transcription start and end sites in prokaryotes and Viruses and underscores their potential roles in gene regulation and genome organization. Analysis of intraspecies variation shows elevated substitution burden in IR spacers and relative conservation of IR arms, particularly near transcriptional terminators. Through a comprehensive overview of the distribution and characteristics of IRs in a wide array of organisms, this largest-scale analysis to date sheds light on the functional significance of perfect IRs, their contribution to genomic instability, and their evolutionary impact across the tree of life.

Significance Despite the importance of inverted repeats (IRs) in regulation and biological function, studies examining their properties across the three domains of life and viruses remain limited. Here, we analyze IRs across thousands of complete genomes and show that they exhibit distinct genomic features and sequence compositions across all domains, while being strategically positioned at key regulatory elements, such as transcriptional terminators. Comparison with shuffled controls reveals that IRs are more abundant than expected by chance across all four domains. Furthermore, intraspecies variation analysis of multiple bacterial pathogens shows that certain species harbor a disproportionate burden of IR polymorphisms. Finally, while IR arms are generally conserved, spacer regions accumulate significantly more single-nucleotide variants than expected by chance.

## Introduction

DNA is the carrier of genetic information used by most organisms. Since the discovery of the DNA double-helical structure (i.e. B-DNA) over 70 years ago, where two antiparallel strands intertwine, many different types of alternative conformations (i.e. non-B DNA) that differ from the classic B-DNA conformation have been discovered. These non-B DNA structures play a wide variety of biological roles in genomic DNA organization, replication, transcription, and recombination and may provide positive selection advantages that make certain non-B DNA motifs conserved (Kaushik et al. 2016; Makova and Weissensteiner 2023). On the other hand, many types of non-B DNA structures are intrinsically mutagenic and therefore provide driving forces for genome evolution and disease development (Georgakopoulos-Soares et al. 2018; Cheloshkina and Poptsova 2019; Wang and Vasquez 2022).

**Fig. 1.**
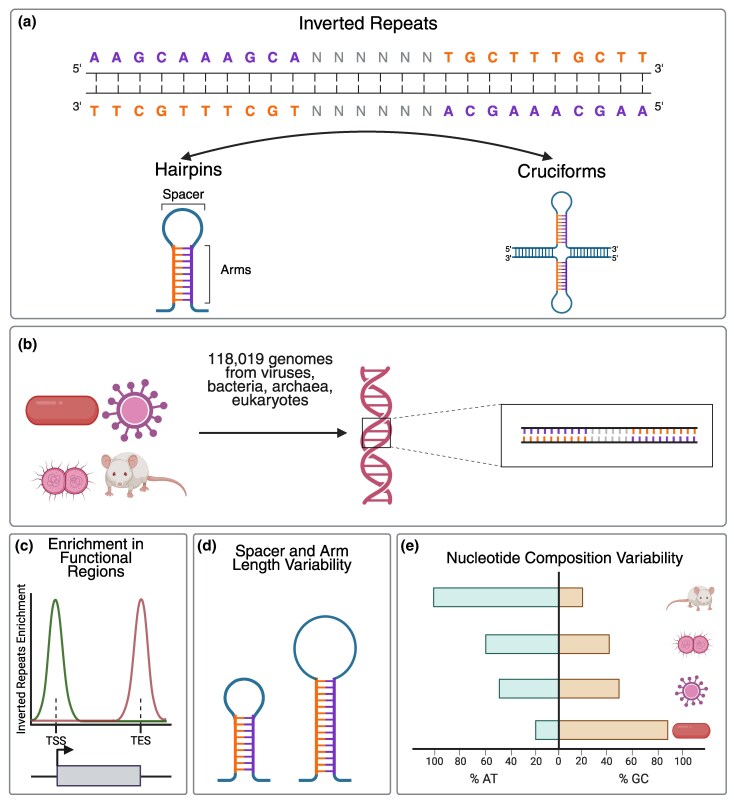
Schematic representation of the investigation of IRs across the tree of life in this study. (a) Schematic representation of an inverted repeat. Colored base-pairs denote the IR arms, and the “N” letters represent the intervening spacer region. Formation of hairpin and cruciform structures can occur at IRs. (b) IR identification was performed across 118,019 complete organismal genomes, including Viruses, Bacteria, Archaea, and Eukaryota. (c) We observed enrichment of IRs relative to functional genomic loci, including transcription start sites and transcription end sites. (d, e) We found that biophysical properties and the nucleotide composition of IRs, including spacer and arm length, influence their frequency across taxa. Created in BioRender. Georgakopoulos-Soares, I. (2026) https://BioRender.com/mwt6x4v.

Alternative DNA conformations can be inferred from the primary nucleotide sequence. One of these conformations is the hairpin/cruciform DNA structure, which can form at inverted repeat (IR) sequences or palindromic motifs (Bikard et al. 2010; Lu et al. 2015; Bacolla et al. 2016; Buisson et al. 2019) (Fig. 1a). In these instances, double-stranded DNA adopts hairpin conformations, with a single DNA strand with two symmetric sequences undergoing intrastrand base pairing to form hairpin or cruciform structures consisting of two complementary arms separated by a spacer loop (Fig. 1a). Physiological processes such as DNA transcription and replication, which involve the opening of double-stranded DNA into a single-stranded state, generate negative supercoiling, which can facilitate hairpin and cruciform formation (Rosche et al. 1995; Azeroglu et al. 2014).

The biophysical properties of IRs impact the likelihood of hairpin/cruciform structure formation. Stable hairpin/cruciform formation is promoted by longer IR arms, while mismatches in the hairpin arms and longer spacer lengths reduce the likelihood of stable structure formation (Nag and Petes 1991; Sinden et al. 1991; Lobachev et al. 1998; Nasar et al. 2000; Rentzeperis et al. 2002). Additionally, the GC content of the stem is associated with increased thermodynamic stability of the DNA hairpin/cruciform (Woodside et al. 2006; Buisson et al. 2019), and the cellular environment contributes to the likelihood of hairpin formation (Tan and Chen 2008).

IRs contribute to genetic instability across prokaryotic and eukaryotic organisms (Lindsey and Leach 1989; Gordenin et al. 1993; Leach 1994; Nag and Kurst 1997; Lobachev et al. 1998, 2007; Zhou et al. 2001; Butler et al. 2002; Tanaka et al. 2002; Achaz et al. 2003). In particular, maintaining long IRs in vivo poses a significant challenge (Leach 1994; Nag and Kurst 1997; Lobachev et al. 1998). In the human genome, IRs serve as mutational hotspots, accumulating an excess of both germline and somatic mutations (Lu et al. 2015; Bacolla et al. 2016; Zou et al. 2017; Georgakopoulos-Soares et al. 2018, 2022c; Buisson et al. 2019; Guiblet et al. 2021; Langenbucher et al. 2021). As a result, the vast majority of IRs are less than 100 base-pairs (bps) long (Kurahashi and Emanuel 2001; Wang and Vasquez 2006). Longer IR sequences do exist in the human genome, particularly in the X and Y chromosomes, but they tend to contain multiple mismatches (Warburton et al. 2004). Long (>500 bp), AT-rich IRs with mismatches have been associated with gross chromosomal rearrangements in the human genome (Kurahashi and Emanuel 2001).

The rapid increase of available genomic data in recent years provides a unique opportunity to study genome structure and composition comprehensively across a wide range of organisms from different taxa of the tree of life. In the coming years, the number of complete organismal genomes is expected to increase exponentially and encompass a significant proportion of the genetic diversity present in nature. For example, the Earth BioGenome Project aims to sequence the genomes of all eukaryotic species within the next 10 years (Lewin et al. 2022). Analyzing the diversity of organismal genomes is essential for uncovering biological insights and has far-reaching implications for advancing our understanding of evolutionary history, unraveling the complexities of functional genomics, and improving our understanding of human health and disease. Previous research on 1,565 bacterial genomes revealed that, for IRs with arm lengths of six bps or longer, the highest and lowest mean frequencies were observed in the phylum Tenericutes (also known as Mycoplasmatota) and the class Alphaproteobacteria, respectively (Porubiaková et al. 2023). Another study examining promoters from 1,180 organismal genomes found an enrichment of IRs in the promoters of prokaryotic organisms (Yella and Vanaja 2023). IRs have also been systematically characterized in chloroplast and mitochondrial genomes (Brázda et al. 2018; Cechová et al. 2018).

Functional roles for IRs have been uncovered (Bikard et al. 2010; Brázda et al. 2011; Grechishnikova and Poptsova 2016). For example, IRs are found at replication origins in prokaryotes, eukaryotes, and viruses (Pearson et al. 1996; Leung et al. 2005) and play diverse roles in gene expression regulation. In gene promoters, they act as inhibitory elements and modulate transcription termination, but at the RNA level, they influence steady-state mRNA expression and stability in 3′ untranslated regions (de Hoon et al. 2005; Chen et al. 2013; Miura et al. 2018; Brázda et al. 2020; Fleming et al. 2020; Siegel et al. 2022; Georgakopoulos-Soares et al. 2022a, 2022c). In prokaryotes, IRs are pivotal structures in rho-independent transcription termination (von Hippel 1998; Kingsford et al. 2007). Additionally, research has identified associations between specific proteins and hairpin or cruciform structures (Brázda et al. 2011). Despite these findings, no comprehensive study to date has systematically characterized IRs across organisms throughout the entire tree of life.

Here, we perform a comprehensive investigation of perfect IRs (without mismatches in the arms, with minimum arm lengths of 10 bps, and with spacer lengths up to eight bps) across 118,019 complete organismal genomes for the domains of life and for Viruses. We report a total of 33,558,920 IRs and find that the density of IRs per genome varies significantly among different phyla. Viruses and Bacteria harbor the highest density of IRs relative to their genome size, whereas Eukaryota and Archaea have the lowest. Genome size is positively correlated with IR density in Archaea, Eukaryota, and Viruses. Nevertheless, the density of IRs per genome varies significantly among different phyla. We also find that the biophysical properties of IRs are highly biased between taxonomies. Eukaryotic IR arms are highly enriched for repetitive AT-rich sequences. Bacteria show a preference for spacers of four bps, whereas Eukaryota give rise to no-spacer perfect palindromes due to the inherently repetitive organization of eukaryotic genomes. Finally, we find that IRs are preferentially positioned upstream of transcription start sites (TSSs) and downstream of transcription end sites (TESs), and Bacteria have a strong preference (exceeding 12-fold enrichment) for IRs downstream of TESs. We conclude that perfect IRs display large discrepancies in abundance between organismal genomes, have notable biophysical differences between taxonomies, and are preferentially located in functional elements.

## Results

### Identification of Perfect IRs Across 118,019 Organismal Genomes

To determine the frequency of long, perfect IRs in nature across organismal genomes, we used 118,019 complete organismal genomes spanning the tree of life for the genome-wide prevalence of IRs, consisting of 49,191 bacterial, 67,654 viral, 687 archaeal, and 487 eukaryotic genomes, respectively (Tables S1 and S2). We focused on perfect IRs, those without mismatches in the arms, as they are more likely to form hairpin/cruciform structures. We used arm sizes longer than nine bps and spacer lengths up to eight bps. These limits were used because IRs with short arms and longer spacer loops are less likely to fold into hairpin/cruciform structures (Nag and Petes 1991; Sinden et al. 1991; Nasar et al. 2000; Rentzeperis et al. 2002).

**Fig. 2.**
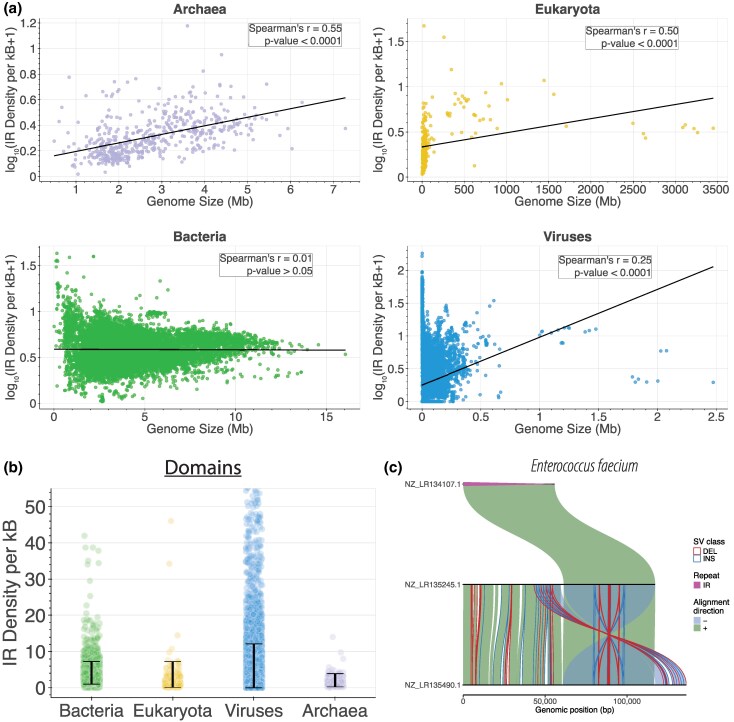
Characterization of IRs in 118,019 organismal genomes across the three domains of life and viruses. (a) Association between the genome size and the proportion of the genome covered by IRs, presented separately for the three domains of life and Viruses. (b) Average IR density per kB for organisms in the three domains of life and Viruses. The *y*-axis is capped at 55 IR per kB. Error bars represent 95% quantile-based confidence intervals. (c) Multiple sequence alignments of three distinct *Enterococcus faecium* plasmids found in GCF_900635415.1 (plasmid NZ_LR134107.1), GCF_900639415.1 (plasmid NZ_LR135245.1), GCF_900639715.1 (plasmid NZ_LR135490.1), RefSeq assembly accessions, respectively. The large inverted repeats are illustrated in purple, while the deletions and insertions are in red and blue, respectively.

We mapped IR sequences across the genomes of each organism for which sequencing data were available, creating a comprehensive genome-wide dataset with species from diverse taxa across the tree of life. We found 33,558,920 IR-forming sequences. Upon examining the IR densities across the distinct taxonomic partitions, we note that Bacteria exhibit the highest average IR density of 3.14 IR bps per kilobase (kB), followed by Viruses (1.93 IR bps per kB) and Eukaryota (1.91 IR bps per kB) (Fig. 2a, b). Strikingly, out of 67,654 examined Viruses, 48.57% (*n* = 32,860) exhibited no IRs with the aforementioned parameters. We observed remarkable variation in the density of IRs across organismal genomes, with densities ranging from 0 IR bps per kB to 180.33 IR bps per kB occurring in *Kummerowia striata partitivirus*, and a median density of 1.306 IR bps per kB (Figs 2b and  S1).

We were interested in finding the longest IRs present in organismal genomes; therefore, we investigated whether there are IRs of hundreds or thousands of bps in any of the studied genomes. Although rare, we found several IRs with arm lengths of hundreds or tens of thousands of bps (Table S3; Fig. S2). In particular, we identified large IRs across multiple bacterial species. Notably, three large IRs were detected on three distinct yet closely related plasmids from *Enterococcus faecium*. One plasmid consisted entirely of a single large IR and aligned perfectly with a region of the second plasmid, while both were homologous to the third. Remarkably, the insertions and deletions observed within the IR regions among these plasmids occurred symmetrically, thereby preserving the IR structure (Fig. 2c). However, IRs with perfect arms of extreme lengths were not identified in *Homo sapiens*, with the longest IR detected having arms of 140 bp (Table S4). We conclude that IRs of arm lengths of thousands of bps without mismatches are almost exclusively found in bacteria.

### Discrepancies in IR Frequencies in Organismal Genomes Across the Tree of Life

We investigated IRs across the three domains of life and Viruses separately. Notably, we found that the proportion of the genome covered by IRs is not strongly correlated with genome size in Viruses (Spearman correlation *r* = 0.25, *P*-value < 0.0001). For Archaea (Spearman correlation *r* = 0.55, *P*-value < 0.0001) and Eukaryota (Spearman correlation *r* = 0.5, *P*-value < 0.0001), this proportion is positively correlated with genome size (Fig. 2a). We also observed that Eukaryota display a high level of repetitiveness in IR arm sequences and are enriched for AT-rich sequences, which are also associated with tandem repeats (Chantzi and Georgakopoulos-Soares 2025). Finally, in Bacteria, we found no correlation between IR genome coverage and genome size (Fig. 2a, Spearman correlation r = 0.01, *P*-value > 0.05). Interestingly, we observed significant differences in the frequency of perfect IRs between Archaea, Eukaryota, Bacteria, and Viruses. Bacteria and Viruses exhibited the highest genomic density of IR with an average density of 3.14 and 1.93 IR per kB, respectively, whereas Eukaryota and Archaea displayed the lowest IR densities per genome with 1.91 and 1.23 IR per kB, respectively (Fig. 2b). These findings suggest differences in the frequencies of IRs associated with taxonomic groups and genome size.

### Substantial Differences in the IR Spacer and Arm Length Distributions Among Taxa

**Fig. 3.**
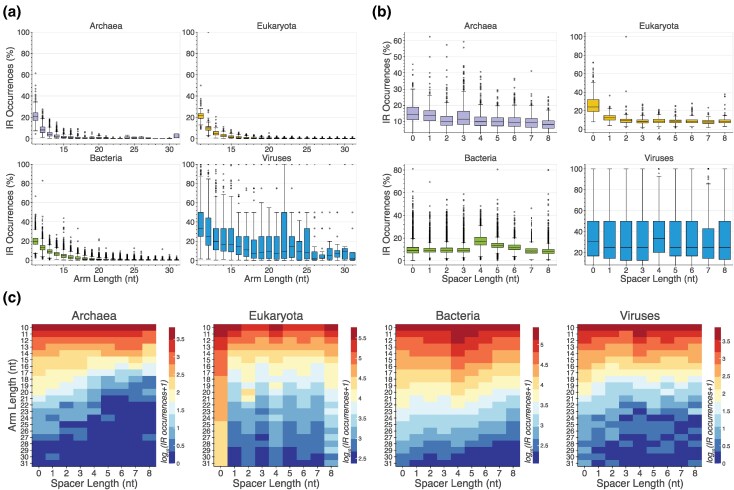
Distribution and density of IR types across taxonomic subdivisions. (a) Percentage (%) of IRs as a function of arm length within each species across the three domains of life and Viruses. (b) Percentage (%) of IRs as a function of spacer length within each species across the three domains of life and Viruses. (c) Heatmaps showing the occurrences of IRs as a function of arm and spacer length combinations.

The biophysical properties of IRs, including the spacer and arm lengths, as well as the nucleotide composition of the hairpin arms, influence the likelihood of hairpin/cruciform formation and stability (Nag and Petes 1991; Varani 1995). Therefore, we examined the effect of these biophysical properties on the frequencies of IRs across taxonomic subdivisions. We observed that across taxonomic groups, the largest number of IRs have short arm lengths (Fig. 3a), which is expected since the probability of finding short complementary sequences by random chance is higher than for longer sequences. In contrast, we find that the number of IRs identified varies significantly for different spacer lengths between various taxonomic groups. In Eukaryota, IRs are predominantly found with zero spacer lengths, whereas in Bacteria, the largest number of IRs have spacer lengths of four bps, whereas in Viruses, there is a preference for spacer lengths of zero or four bps (Fig. 3b, c).

**Fig. 4.**
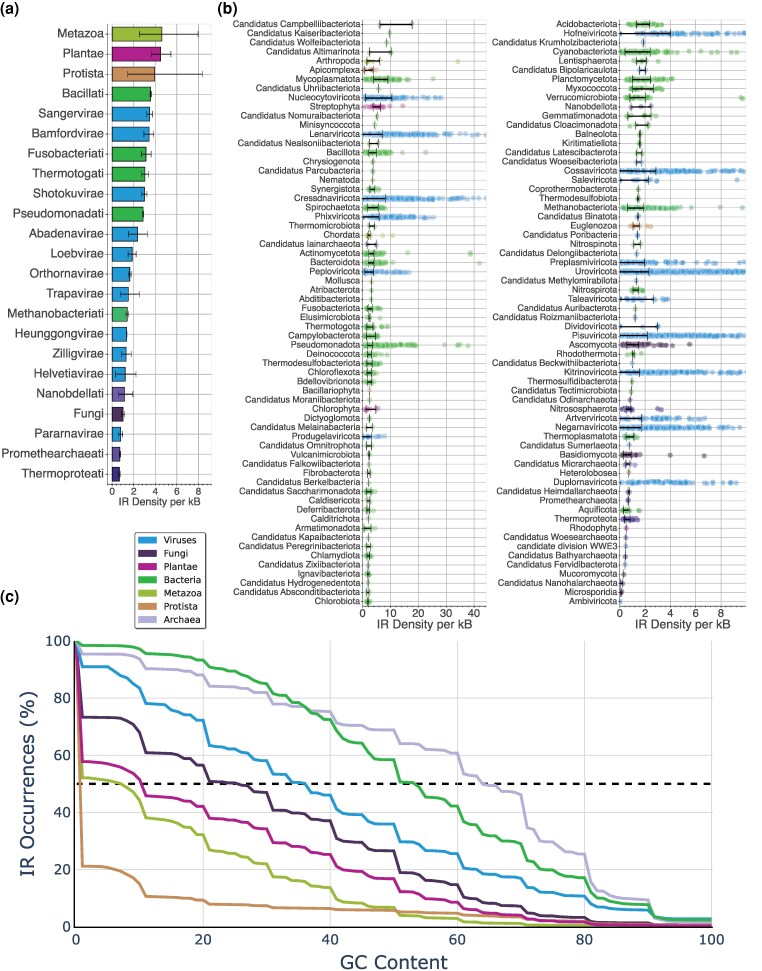
Genomic density of IRs in organismal genomes across the tree of life. (a) Average IR coverage across organismal genomes belonging to the same kingdom. The color represents the three domains and Viruses to which each of the kingdoms belongs. (b) Jitter plots showing the IR density per kB across different phyla. The color represents the kingdom to which each of the phyla belongs if eukaryotic, or the domain of life for prokaryotes and Viruses. Error bars represent the 20th–80th percentile range of IR density across species within each phylum. (c) IR occurrences as a function of the total IR GC-content in the arm sequence. Results are shown at the kingdom and domain levels.

Next, we investigated differences in the genomic density of IRs across kingdoms and phyla. The kingdoms Protista, Metazoa, and Plantae exhibited the highest IR density per kB, with 4.8, 4.55, and 4.22 IR per kB, respectively. The next most IR dense were bacterial kingdoms Bacillati, Fusobacteriati, and Pseudomonadati with 3.67, 3.28, and 3.10 IR per kB, respectively, followed by Viral kingdoms Shotokuvirae with 3.03 IR per kB, Sangervirae with 3.00 IR per kB, and Loebvirae with 2.62 IR per kB (Fig. 4a). In contrast, Thermoproteati exhibited the lowest IR density with 0.64 IR per kB. These findings indicate substantial differences in the IR density across kingdoms, even within the same domain. At the phylum level, the two phyla with the highest density of IRs were both bacterial, namely *Candidatus campbelliibacteriota* and *Candidatus kaiseribacteria*, with an average IR density of 12.00 and 9.7 IR density per kB, respectively (Fig. 4b). Additionally, Apicomplexa, a protist phylum, exhibited a high IR density of 6.70 IR per kB. High IR densities were also observed in *Mycoplasmatota*, *Candidatus uhriibacteriota, Nucleocytoviricota*, and *Streptophyta*, which belonged to Bacteria, Plants, and Metazoa (Fig. 4b). These findings underscore the substantial variability in the IR density across domain, kingdom, and phylum levels.

Next, we examined the contribution of GC content to the number of IRs detected per organism across kingdoms and phyla. We investigated the genomic density of IRs as a function of GC content. We observed that in Eukaryota, there is a steep decline in the number of IRs detected as we increase the percent of GC content in the arm (Fig. 4c). On the other hand, such a sharp decline is not observed in Viruses, Bacteria, or Archaea, indicating that the vast majority of eukaryotic IRs are GC-depleted. Thus, when excluding AT-rich IRs, Eukaryota have the lowest IR density compared with Archaea, Bacteria, and Viruses, and this pattern is consistent at the kingdom level (Fig. 4c). Specifically, when examining IRs with a GC content of at least 10%, we find that the highest genomic density is observed in Bacteria, followed by three viral kingdoms, namely Sangervirae, Shotokuvirae, and Loebvirae. This trend is also observed for a GC content threshold of 20% (Fig. S3). This result is only partially accounted for by differences in the GC content of organismal genomes, indicating a subset of highly prevalent AT-rich and repetitive IRs in Eukaryota. We conclude that repetitive, AT-rich IRs are the most common IRs in Eukaryota, accounting for the vast majority of IR occurrences in Eukaryota, but not in Bacteria, Archaea, or Viruses.

### IRs are Enriched in Eukaryotic, Prokaryotic, and Viral Genomes

**Fig. 5.**
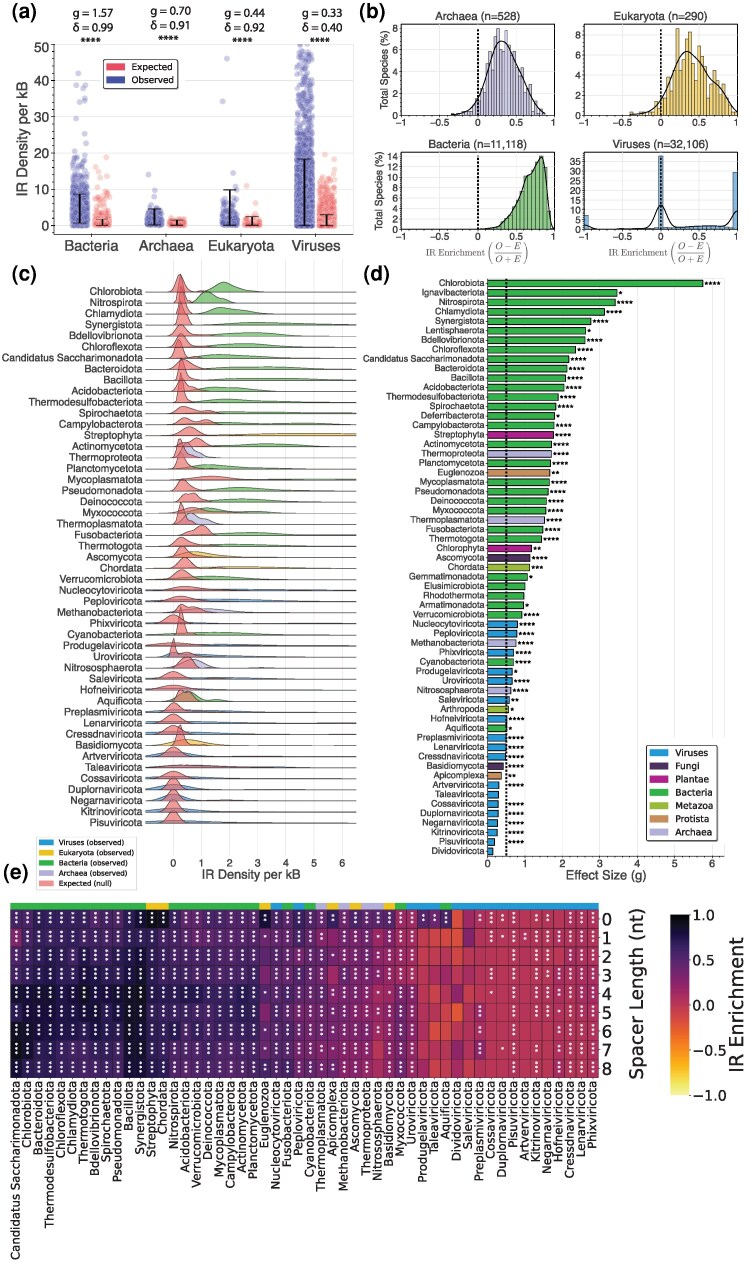
Statistical and effect size assessment of IR enrichment across organismal genomes. (a) Comparison of genome-wide IR density for each species and the dinucleotide-preserving shuffled controls across Eukaryota, Bacteria, Archaea, and Viruses. The *y*-axis is capped at 50 IR per kB. Error bars denote the 95% quantile-based confidence interval. Statistical assessment was performed using a two-sided Wilcoxon signed-rank test and adjusted for multiple comparisons using the Benjamini–Hochberg procedure. Effect sizes (Hedges’ *g* and Cliff's *δ*) were computed to quantify the magnitude of group differences (see Methods). (b) Distribution of IR enrichment across organismal genomes of the three domains of life, including Viruses. The histogram shows the percentage (%) of total species assigned to each individual bin of IR enrichment relative to matched controls; the overlaid curve is the kernel density estimate. (c) Distribution of observed and expected IR density (in red) across phyla, calculated using the kernel density estimation. Only phyla with at least 13 species are shown. (d) Effect sizes (Hedges’ *g*) of IR density in organismal genomes versus dinucleotide-preserving shuffled controls across phyla, displayed in descending order. Bar colors indicate the kingdom for eukaryotes and the domain for all other groups. Bars above the dotted black line correspond to higher-than-medium effect sizes. Statistical assessment was conducted using a two-sided Wilcoxon signed-rank test, adjusted for multiple comparisons using the Benjamini–Hochberg procedure. Adjusted *P*-values are indicated as * for *P* < 0.05, ** for *P* < 0.01, *** for *P* < 0.001, and **** for *P* < 0.0001. (e) Average IR enrichment (O − E)/(O + E), where O is the observed and E the expected IR density, respectively, across phyla, partitioned by spacer length. Each cell represents a (phylum, spacer length) pair. *P*-values correspond to the comparison between observed and expected IR density of species within that phylum for each spacer length, calculated using a two-sided Wilcoxon signed-rank test and adjusted for multiple comparisons using the Benjamini–Hochberg procedure. Adjusted *P*-values are indicated as * for *P* < 0.05, ** for *P* < 0.01, and *** for *P* < 0.001.

We examined whether IRs are more abundant than would be expected by chance. To this end, for each organismal genome in our database, we generated matched shuffled genomic sequences that preserve the local dinucleotide structure in every kilobase. We report that all three domains of life and Viruses exhibited a significantly higher genomic IR density than expected by chance. The highest effect size was observed in Bacteria (Hedges’ *g* = 1.57), followed by Archaea (Hedges’ *g* = 0.70) and Eukaryota (Hedges’ *g* = 0.44) (Fig. 5a). On the other hand, Viruses, on the domain level, exhibited the lowest enrichment, with 35% of the total population lacking IRs in real and synthetic controls. Additionally, the vast majority of prokaryotic and eukaryotic species displayed a positive enrichment of IRs (Fig. 5b).

At the phylum level, bacterial phyla exhibited significant shifts from the null distribution, indicating a strong enrichment of IRs relative to dinucleotide-preserving controls. Moreover, across the bacterial phyla, the observed IR density displayed substantially elevated variance compared with matched synthetic sequences. The latter were comparatively more concentrated, suggesting that IR expansions across lineages cannot be attributed solely to the local genomic dinucleotide structure. Such differences were not observed in archaeal phyla, which displayed smaller, more centralized shifts from the null distribution (Fig. 5c). When ordered by their respective effect sizes, the top 15 phyla originated from the bacterial domain; the highest effect size was observed in the Chlorobiota phylum (Hedges’ *g* = 5.75), a group of green sulfur, photoautotrophic bacteria. In terms of ranking, these top phyla achieved maximal separation from their respective control groups (Cliff's *δ* = 1; two-sided Wilcoxon signed-rank test, adjusted *P*-values < 0.05). In fact, for the top phyla, even when disregarding the natural pairing between the real and the synthetic genomes, the former stochastically dominated the latter (unpaired Cliff's *δ* > 0.9; two-sided Mann–Whitney *U* test, adjusted *P*-values < 0.05), suggesting qualitatively distinct distributions (Fig. 5c and d). The vast majority of eukaryotic species displayed positive IR enrichment, with eukaryotic phyla exhibiting large effect sizes. For instance, Streptophyta demonstrated the highest effect size among the studied eukaryotes (Fig. 5d). When partitioning the IRs according to their spacer length, we observe that most prokaryotic and eukaryotic phyla are enriched across all spacer lengths, often with high enrichment values. Aquificota was the only bacterial phylum that did not exhibit significant differences, except for perfect palindromic IRs. A common theme among eukaryotic phyla is a pronounced enrichment for perfect palindromic repeats (spacer length = 0) (Fig. 5e). These results suggest that IRs are ubiquitous across all domains of life, with the most abundant in bacterial lineages.

### Biophysical Properties of IRs Across Taxonomic Groups in the Tree of Life

**Fig. 6.**
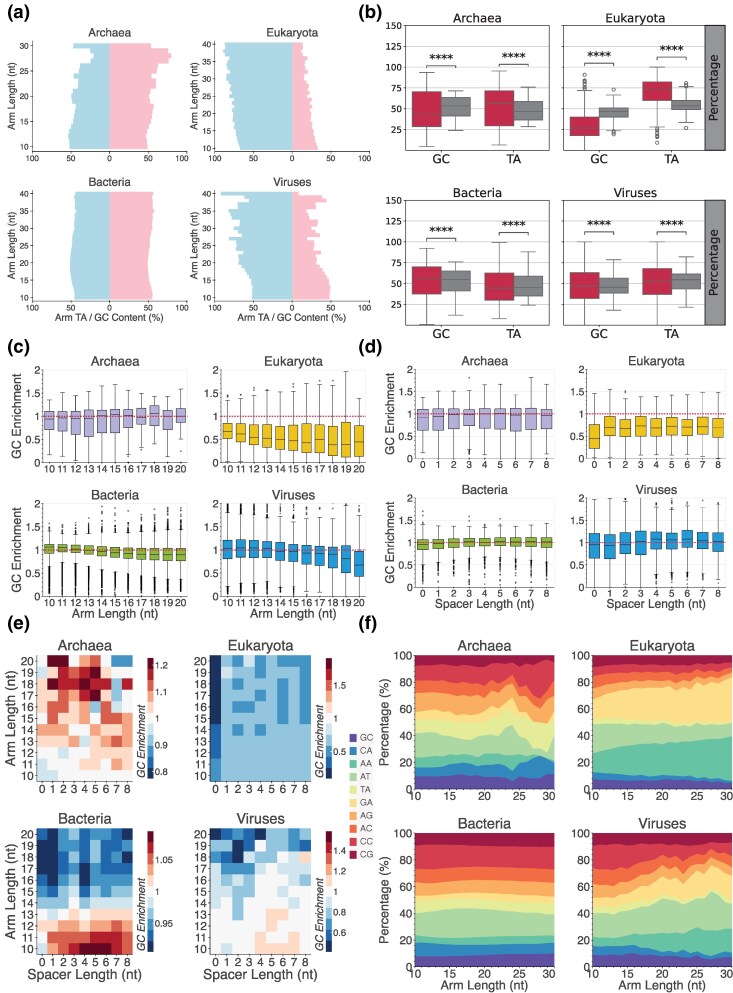
Characterization of IRs by their biophysical properties and nucleotide composition. (a) Comparison of GC and AT content in IR arms across genomes as a function of arm length. (b) GC/AT content in IR arms compared with the genome-wide GC/AT content. Differences were assessed using a two-sided Wilcoxon signed-rank test, with *P*-values adjusted for multiple comparisons using the Benjamini–Hochberg procedure. Adjusted *P*-values are represented as * for *P* < 0.05, ** for *P* < 0.01, *** for *P* < 0.001, and **** for *P* < 0.0001. (c, d) GC ratio in arms versus genome-wide GC-content as a function of **c**. arm and **d**. spacer length. (e) Heatmaps showing GC enrichment of IRs across combinations of arm and spacer lengths. (f) Percentage of the different dinucleotides in arms as a function of arm length.

Next, we investigated the nucleotide composition of the arms relative to that of each organism's genome across the three domains of life and Viruses. We observe that in Eukaryota, there is higher AT content in the IR arms. Viruses also displayed high AT content, which increased with IR arm length (Fig. 6a). In contrast, such differences were not observed in Bacteria and Archaea, with Archaea showing an excess of GC relative to AT in the IR arms (Fig. 6a). When comparing GC content of IR arms to genome-wide background levels within each species, we observe a significant depletion of GC content in Eukaryota and Archaea (two-sided Wilcoxon signed-rank test, *P* < 0.0001 and *P* < 0.0001, respectively). However, this depletion is biologically meaningful only in Eukaryotes, where the effect size is substantial (Cliff's *δ* = −0.74). In contrast, Bacteria show a significant excess of GC-rich IRs relative to the background (two-sided Wilcoxon signed-rank test, *P* < 0.0001; Fig. 6b), but the corresponding effect size indicates a negligible overall effect.

When performing the same analysis separated by arm and spacer length, we found that the main deviation is in Eukaryota, in which IRs without spacers tend to be disproportionately AT-rich compared with the genomic background (Fig. 6c, d). Further stratification by IR arm length reveals that, in Bacteria, GC-enrichment displays a unimodal distribution for short IR arms (<12 bp; Hartigan's dip test, *P* > 0.05), whereas clear bimodality emerges for longer IRs (≥12 bp; Hartigan's dip test, *P* < 0.0001). Notably, for IR arms of 10 and 11 bp, GC enrichment shows a small-to-medium effect size (Cliff's *δ* = 0.30 and 0.33, respectively) (Figs S4–S5). Additionally, the GC-enrichment signal is stronger for IRs with small arms and spacer lengths between 4 and 6 bases (Fig. 6e).

Next, we investigated the dinucleotide content of the arms as a function of IR length. We found that IRs with longer arm lengths have a disproportionate AT/TA content in Eukaryota (Fig. 6e, f). Therefore, we conclude that the biophysical properties of IRs, which influence the likelihood and stability of hairpin/cruciform formation, exhibit distinct properties across the three domains of life and Viruses.

### IRs are Inhomogeneously Distributed at Functional Genomic Elements and are Positioned Relative to Transcription Start Sites (TSSs) and Termination End Sites (TESs)

Previous work has shown that IRs are inhomogeneously distributed in the human genome and have several functional roles in several other organismal genomes (von Hippel 1998; de Hoon et al. 2005; Chen et al. 2013; Miura et al. 2018; Brázda et al. 2020; Fleming et al. 2020; Siegel et al. 2022; Georgakopoulos-Soares et al. 2022a, 2022c). Therefore, we investigated whether the distribution of IRs is heterogeneous in functional genomic regions and whether they are preferentially positioned relative to TSSs and TESs in the genomes of organisms belonging to different taxonomic clades.

**Fig. 7.**
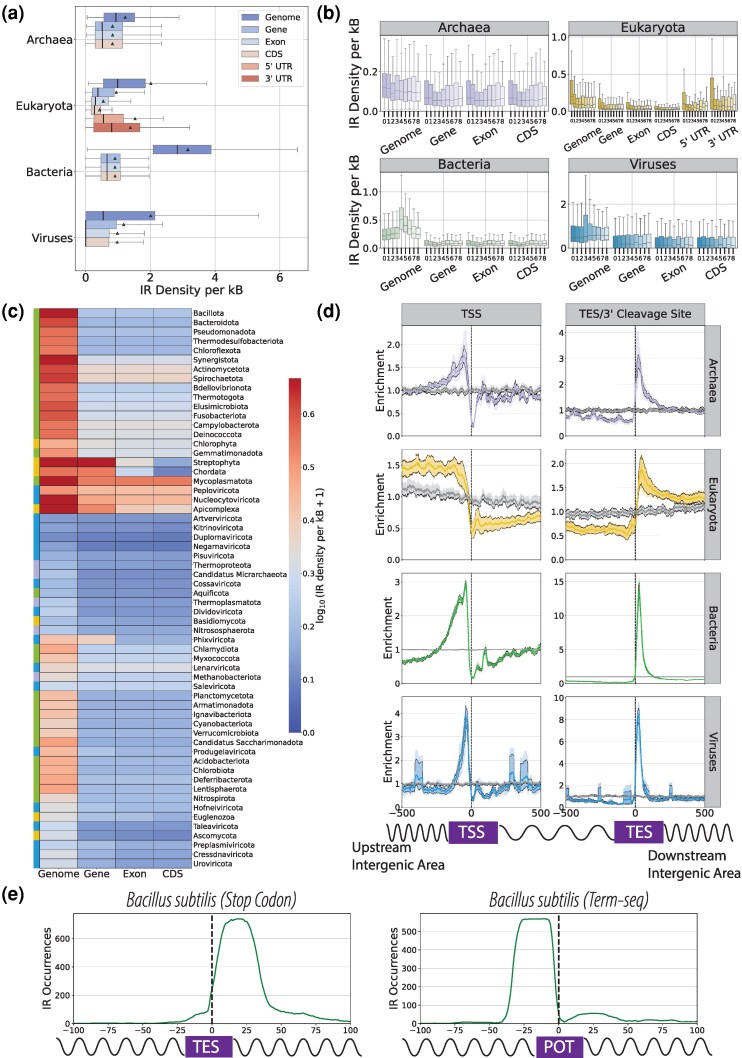
Examination of the topography of IRs across organismal genomes belonging to different taxonomies in the tree of life. (a) IR density across genomic subcompartments for the three domains of life and Viruses. (b) IR density across genomic subcompartments for the three domains of life and Viruses for the different spacer lengths. For each compartment, the spacer lengths appear in increasing order from 0 to 8 base pairs. (c) Heatmap of the IR density across phyla at the CDS, exonic, genic, and genome-wide regions. Color bars on the left indicate the three domains of life and Viruses, as well as the six kingdoms to which the different phyla belong. (d) IR distribution across the three domains of life and Viruses, relative to the translation (prokaryotic)/transcription (eukaryotic) start sites (TSS) and translation (prokaryotic) end site (TES)/3′-cleavage sites (eukaryotic), represents the positional bias of IRs at each locus within the 1 kB window in comparison to the window average. Confidence intervals represent the 2.5% lowest and 97.5% highest percentiles from Monte-Carlo simulations with replacement on the species (inner layer) and on the family (outer layer) taxonomic level (*N* = 1,000). The gray lines represent the control group, which was generated by detecting IRs in the shuffled genomes while preserving the local dinucleotide sequence composition. (e) IR distribution in *Bacillus subtilis str. 168*, relative to the translation end site (TES) (left) and the point of termination (POT) (right).

First, we examined the distribution of IRs across functional genomic sub-compartments for each genome, including genome-wide, genic, exonic, 5′ UTR, 3′ UTR, and coding sequence (CDS) regions. We observed that, across the three domains of life and Viruses, IRs are predominantly concentrated in intergenic regions (Fig. 7a). Notably, Bacteria exhibit, on average, the highest genome-wide IR density of 3.14 IR bps per kB, followed by Viruses and Eukaryota. Despite the Viruses exhibiting a higher average IR density than Archaea, their median is the lowest amongst all four domains (Fig. 7a). Upon partitioning the various IRs by their spacer length, we noted significant differences in IR densities across the genomic compartments (Fig. 7b). In Eukaryota, the exonic and CDS regions displayed low IR density for all spacer lengths, whereas a general propensity for perfect palindromes with zero bp spacer sequences is observed in genic regions. Additionally, when comparing the IR distributions across eukaryotic kingdoms, the intronic IR densities appeared consistently elevated compared with the exonic ones (Fig. S6). This leads us to conclude that IRs are favorably positioned within intronic regions and 3′ and 5′ UTR regions. In Bacteria, the vast majority of IRs are positioned within intergenic regions, with 4 bp being the most prevalent spacer length. In viral genomes, intergenic IR density is the highest (Fig. 7b). When comparing individual phyla, we consistently observed that intergenic regions display the highest density of IRs across phyla, whereas there are larger differences in densities at genic, exonic, and CDS regions between eukaryotic phyla (Figs 7c and S6–S7). These findings indicate that intergenic regions are most enriched for IRs across taxonomic groups, which could be due to the RNA polymerase stalling potential of hairpin and cruciform structures in genic regions (Hein et al. 2014).

Next, we investigated whether IRs are differentially distributed relative to annotated TSSs and 3′-cleavage sites (eukaryotic organisms), and, analogously, for prokaryotic and viral accessions, we used the translation start and end sites, hereafter denoted as TSS and TES, respectively. We observed that across the three domains of life and Viruses, there is consistent enrichment of IRs in the 100 to 200 bp regions upstream of the TSSs, with peaks located at approximately −50 bp in Archaea, Bacteria, and Viruses, and at −150 bp in Eukaryota. IR densities drop dramatically at the TSSs (Figs S8–S10). IR motifs are also enriched in the regions downstream of the TESs in all domains and Viruses. IR densities exhibited very narrow and high peaks in Archaea, Bacteria, and Viruses within the tight regions of +50 bp to +80 bp downstream of the translation stop codon (Figs 7c and S10–S12), suggesting that they play roles in transcription initiation and termination across organismal genomes. Of particular note, the distribution of IRs after the translation stop codon is 13-fold more enriched in Bacteria (Fig. 7c) than in other domains and Viruses, which can be attributed to their role in rho-independent transcription termination (d’Aubenton Carafa et al. 1990; von Hippel 1998). We found that the enrichment of IRs after the TESs in Bacteria is present across all phyla, albeit with varying effect sizes (Figs 7c, d, and S8 and S10), indicating a general, ubiquitous bacterial mechanism of gene regulation. It is also noteworthy that the same distribution is present in Archaea, although with lower enrichment levels (Figs 7c, d and S11) and significant differences between archaeal phyla. Hitherto, we relied on largely computationally predicted genic annotations. To further elucidate the role of IRs in bacterial termination, we leveraged experimental Term-seq data from the *Bacillus subtilis strain 168* (Kosiński et al. 2025). We noted that IRs preferentially accumulated downstream of computationally predicted translation stop codons and upstream of points of termination (POT), as inferred from the Term-seq data (Fig. 7e).

We segmented the IR counts into nine distinct groups characterized by the spacer length of the IR and studied the distribution of IR occurrences across the 1-kB window around the TSS and TES for each spacer length. Furthermore, we partitioned the analysis into protein-coding and non-coding genes across the three domains of life, including viruses. In prokaryotic species, the protein-coding distribution of IRs is enriched upstream of the TSS and downstream of the TES (Fig. S13). Notably, in prokaryotes, the non-coding distribution of IRs is enriched downstream of the TSS; hence, the putative hairpin loop lies within the genic region and not upstream of the TSS. This discrepancy between non-coding and protein-coding IR positioning could be attributed to the different translational machinery deployed by the cell between the transcription of protein-coding versus non-coding RNAs, as well as the secondary structure of non-coding RNAs containing multiple hairpin loops. This phenomenon is not observed in eukaryotic organismal genomes, while in viruses, the enrichment hotspots are dispersed in multiple regions, probably related to the different types of non-coding RNAs (Fig. S13). Additionally, in the TES, there is a higher degree of homogeneity between protein-coding and non-coding RNAs in IR distribution across the four domains. In particular, in bacterial genomes, we found that the majority of IRs consisted of 4 bp spacers. Downstream of TES, we find that the IR hotspot consists primarily of IRs with 4 or 5 bp spacer length (Fig. S13). Experimentally, these have been validated as the most stable forming hairpin loops (Varani 1995), and the stability of the hairpin loop could contribute to rho-independent termination (von Hippel 1998; Kingsford et al. 2007). These findings indicate that IRs are positioned relative to key regulatory elements and may play roles in transcription initiation and termination across diverse organisms.

### Association of Intraspecies Polymorphisms With IRs

Our analysis focused primarily on relatively long, perfect IRs with short spacer lengths because the scale of the analysis was prohibitive to investigate very low arm lengths, as the total occurrences of IRs rise exponentially with respect to the arm length. The mutagenicity of IRs is established by numerous studies. However, the current literature on IR polymorphism across bacterial populations is rather limited. The question that emerges is to what extent perfect IRs are conserved within each species. To investigate this question, we leveraged intraspecific variants in bacterial genomes from our previous work (Chantzi and Georgakopoulos-Soares 2025).

**Fig. 8.**
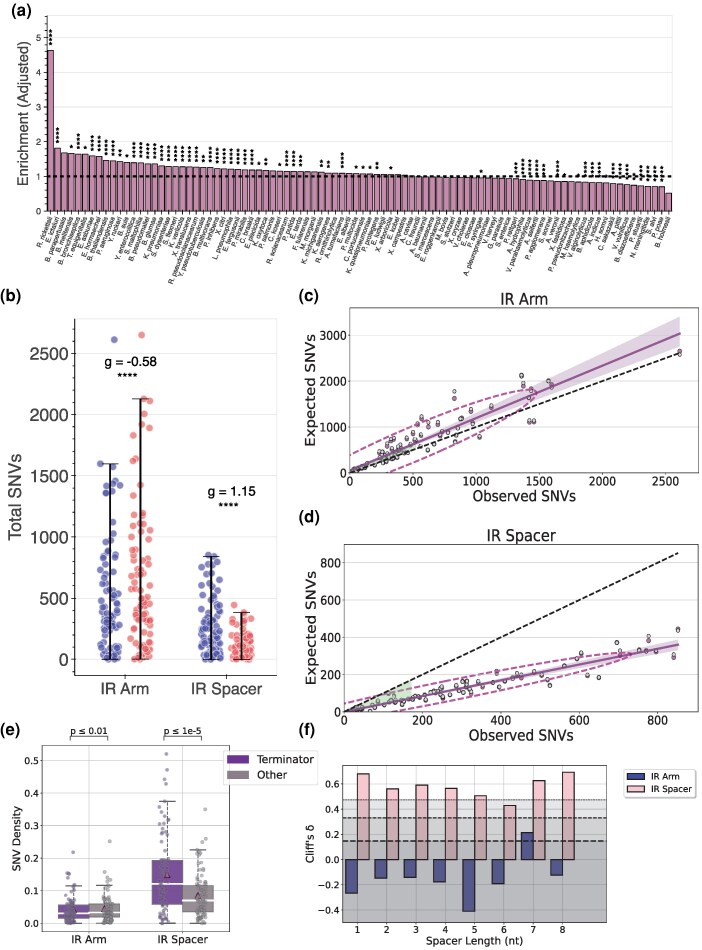
Examination of IR polymorphism across bacterial species. (a) Proportion of the total observed SNVs within IRs with respect to expected SNVs within IRs after adjusting for trinucleotide content composition, on the basis of the genome-wide trinucleotide SNV profile. Results are displayed for *Pseudomonadota* species. Statistical significance was assessed using a two-tailed Fisher's exact test, and the *P*-values were adjusted for multiple hypothesis testing using the Benjamini–Hochberg procedure. (b) Comparison of the total number of observed and expected SNVs within IR arms and spacers, respectively, adjusting for segment length and trinucleotide content composition. Statistical significance was assessed using a two-sided Wilcoxon signed-rank test, and *P*-values were adjusted for multiple hypotheses testing using the Benjamini–Hochberg procedure. Adjusted *P*-values are displayed as * for *P* < 0.05, ** for *P* < 0.01, *** for *P* < 0.001, and **** for *P* < 0.0001. (c, d) Regression of observed against expected SNVs within IR arms and spacers, shown in **c** and **d**, respectively. Purple dots represent the observed SNVs regressed against the expected SNVs after adjusting for the trinucleotide content composition. The confidence interval represents 95%. The gray dots represent the expected SNVs based only on the genome-wide SNV density after adjusting for the length of IR arms and/or IR spacers, respectively. The black line is the 45° angle, which serves as a landmark for the deviation of the observed from the expected occurrences. The ellipses denote the Gaussian KDE between the observed and expected SNV occurrences within IRs after trinucleotide context adjustment. (e) Comparison of the observed SNV density between IRs detected in terminator regions and other genomic contexts within IR arms and spacers, respectively. Statistical significance was assessed using a two-sided Wilcoxon signed-rank test, and *P*-values were adjusted for multiple hypotheses testing using the Benjamini–Hochberg procedure. (f) Cliff's delta effect sizes of the observed SNV density differences between IRs detected in terminator regions and other genomic contexts within IR arms and spacers, respectively, partitioned by spacer length.

First, we examined whether, among the studied organisms, IRs show a propensity for single-nucleotide variant (SNV) accumulation. Indeed, we found that among the studied bacterial species, many exhibited higher SNV densities within IR loci than would be explained solely by their trinucleotide content composition. Notably, *Rickettsia rickettsii*, a gram-negative obligate intracellular bacterium and the causative agent of Rocky Mountain spotted fever (Karpathy et al. 2007), exhibited the highest enrichment of SNVs within IR loci across the 27 strains analyzed. Other bacterial pathogens, such as *E. ictaluri,* also exhibited a high enrichment of SNVs within IRs (Fig. 8a).

In human somatic cells, IRs are found to be mutagenic, and spacer sequences harbor a significantly larger substitution burden than arms (Zou et al. 2017). To examine if IR arms tend to be more conserved than the spacers, we calculated the observed total substitution burden in IR arms and IR spacers separately. Accounting only for the baseline genome-wide substitution density and the genomic length, we report that IR spacers exhibit a larger number of SNV sites than what is expected by chance (two-sided Wilcoxon signed-rank test, *P*-value < 0.0001). To investigate if these results persist through a trinucleotide content adjustment, we repeated this analysis, but this time the estimated substitution burden in IR arms and spacers, respectively, was based on the trinucleotide content composition. After adjusting for the trinucleotide context, we report that the substitution burden in IR spacers remained significantly different from expectation (two-sided Wilcoxon signed-rank test, *P*-value < 0.0001), with a large effect size (Hedges g = 1.14). On the contrary, IR arms appeared significantly depleted (two-sided Wilcoxon signed-rank test, *P*-value < 0.0001) (Fig. 8b). As a next step, we sought to evaluate if the observed signal constitutes a general pattern across the studied bacterial genomes, rather than a subset of species driving the result. And, indeed, when regressing the observed substitution burden in IR arms and spacers, we find that in spacers there is a larger deviation than in the arms, from the expected 45-degree line, suggesting that the IR spacers systematically accumulate a plethora of substitutions than can be explained solely based on the genomic length and the trinucleotide context. Au contraire, such a phenomenon is not present in IR arms, whereas the regression line approximately coincides with the 45-degree line of the first quadrant (Fig. 8c, d).

We hypothesized that, due to the importance of IRs in biological processes such as intrinsic termination, IRs positioned proximal to the terminator region would further amplify the observed discrepancy between arm and spacer SNV density. In fact, when partitioning the IRs into two distinct groups, differentiating between those that occur within 50 bases of the terminator and the rest, the observed SNV density within IR spacers is significantly higher in IRs that occur within terminator regions rather than in different genomic contexts with a strong effect size (Cliff's *δ* = +0.83, two-sided Wilcoxon signed-rank test, *P*-value < 0.0001). However, in IR arms, the pattern was reversed, with arms occurring within terminator regions displaying a significantly lower SNV density compared with those in other genomic contexts, albeit with a moderate effect size (Cliff's *δ* = −0.37, two-sided Wilcoxon signed-rank test, *P*-value < 0.01) (Fig. 8e). We further report that this observation is consistent for the majority of spacer unit lengths (Fig. 8f).

Overall, we find that after adjusting for the local trinucleotide content composition and the IR length, IR spacers harbor a larger amount of substitutions than what is expected by chance alone, whereas IR arms appear to be more conserved, with much less apparent intraspecific polymorphism across different bacterial strains. In addition, the observed polymorphism within IR spacers is pronounced in IRs occurring within terminator regions, highlighting their important regulatory roles in bacterial biological processes such as transcriptional termination.

## Discussion

Here, we examined 118,019 complete organismal genomes to characterize the distribution, biophysical characteristics, and topography of perfect IRs. We observed that the frequency of perfect IRs varies substantially between different organismal genomes and taxonomic groups. Bacteria harbor the highest density of IRs, whereas Archaea have the lowest. However, large discrepancies are observed both between kingdoms and among different phyla within the same domain of life, or Viruses. Such variation may suggest differences in environmental pressures, repair mechanisms associated with IRs, and/or the ability of certain organisms to process these structure-forming sequences in an error-free manner to mitigate the genomic instability associated with these sequences. For instance, replication fork stalling can occur at IRs (Lindsey and Leach 1989; Voineagu et al. 2008). In both prokaryotic and eukaryotic cells, IRs can cause genomic instability, including double-strand breaks, translocations, and deletions (Lobachev et al. 2002; Kurahashi et al. 2006; Narayanan et al. 2006; Lu et al. 2015). We also observed a subset of IRs with arms spanning tens of thousands of bps, which could have resulted from genomic rearrangement events such as inversions (Fried et al. 1991). Previous work has indicated that the presence of IRs is linked to a higher frequency of DNA double-strand breaks and inversions in human cells (Al-Zain et al. 2023); however, it remains to be studied whether similar mechanisms drive the formation of large IRs and whether hairpin structure formation at these large, perfect IRs contributes to genomic instability.

IRs were previously found to be substantially enriched in regions near start codons, stop codons, the ends of genes, 5′-untranslated regions, and promoter regions in *E. coli* (Miura et al. 2018), consistent with our findings. These results suggest a role for IRs in gene expression regulation, potentially providing a selective evolutionary advantage that maintains IRs in these regions despite their intrinsic mutagenicity. In our study, although all domains and Viruses exhibited IR enrichments upstream of the TSSs and immediately downstream of the TESs, Eukaryota showed more variation, with wider enrichment peaks compared with other domains. This may reflect the fact that eukaryotic cells generally have larger genes, longer intergenic regions, and more complex transcriptional control elements in their genomes.

The phyla studied exhibit diverse characteristics and show considerable variation in their IR genomic frequency, biophysical properties, and nucleotide composition. For instance, in prokaryotes, *Pseudomonadota* and *Bacillota* have lower genomic IR densities compared with *Mycoplasmatota*. Notably, *Aquificota*, autotrophs found in extreme environments such as hot springs and sulfur pools, display a significantly lower IR density compared with other bacterial phyla, similar to those of archaeal phyla. This could be due to specific environmental pressures affecting the likelihood of IR formation, as previously demonstrated (Tan and Chen 2008), and potentially negatively impacting the organism's ability to resolve them. The preferential distribution of IRs relative to TSSs and TESs was particularly elevated in Bacteria and Archaea. This may be explained by the role of IRs in rho-independent transcription termination (von Hippel 1998; Kingsford et al. 2007). The presence of dyad symmetry in broader terminator regions halts the transcription process by enabling the dissociation of the ternary elongation complex, causing the RNA polymerase to detach. This phenomenon is widespread across bacterial species, where IRs play a vital role in the intrinsic termination process.

Furthermore, the enrichment of IRs relative to regulatory elements, including TSSs and TESs, indicates that IRs are not merely statistical artifacts but are essential for key regulatory and functional mechanisms within the cell, reinforcing previous research (de Hoon et al. 2005; Kingsford et al. 2007; Chen et al. 2013; Miura et al. 2018; Brázda et al. 2020; Fleming et al. 2020; Siegel et al. 2022; Georgakopoulos-Soares et al. 2022a, 2022c). Additionally, the differences in IR densities between taxa may reflect functional roles specific to individual taxonomic groups, as previously reported (Kingsford et al. 2007; Poggi and Richard 2021; Bowater et al. 2022).

Our analysis used a minimum arm length of 10 base pairs and a maximum spacer length of eight base pairs, consistent with prior observations that shorter arms and longer spacers are less likely to form IRs (Woodside et al. 2006; Zou et al. 2017). Including longer spacers and shorter arm lengths would increase the number of putative IRs detected, but these candidates are biophysically less likely to adopt stable IR structures and may inflate false positives, underscoring the need for parameter-sensitivity analyses in future work. One limitation of our study is that we analyzed only perfect IRs across the tree of life. We deliberately excluded imperfect IRs because arm mismatches substantially reduce the probability of hairpin/cruciform formation (Sinden et al. 1991; Lobachev et al. 1998; Rentzeperis et al. 2002) and, given the vast number of assemblies surveyed, the combinatorial space of mismatched IRs would be prohibitively large to analyze at scale. Although our work did not focus on imperfect IRs, we conducted a case study across several bacterial pathogens. In particular, we showed that certain pathogens, such as *R. rickettsii*, exhibit a disproportionately elevated SNV density within IRs. Additionally, we found that IRs have a pronounced effect on the spacer region rather than on the arms. Future work is required to examine imperfect IRs across organisms spanning the tree of life and their conservation. Another limitation of our work is that our study relies on computational predictions without experimental validation, which may introduce biases or inaccuracies in identifying and characterizing IRs. Experimental studies will be critical for confirming the biological relevance and functionality of the computationally identified IRs and our conclusions.

IRs are dynamic genomic features whose expansion and contraction can be driven by a variety of mechanisms, including replication slippage, unequal crossing over, and transposable element activity. Many IRs coincide with or lie near structured functional elements, such as intrinsic (Rho-independent) transcription terminators in bacteria and hairpin-forming precursors of microRNAs and other ncRNAs, where secondary structure can modulate transcriptional termination, RNA processing, or stability. IRs are also enriched within transposable elements (notably TIR DNA transposons and MITEs), providing abundant inverted sequence tracts that seed hairpins/cruciforms (Feschotte and Pritham 2007). In fact, TIRs, a specific class of IRs flanking DNA transposons, have recently been shown to trigger a spliceosome-independent RNA surveillance mechanism in animals (Zhao et al. 2026). This result further highlights the functional importance of IR secondary structures beyond transcriptional regulation. Future studies of IRs in transposable elements across organismal genomes could reveal their contributions to transposon mobilization mechanisms, structural variation, and host-transposon interactions.

Finally, we acknowledge that sequencing and assembly errors could influence the detection of IRs. Base-calling inaccuracies may disrupt or create short palindromic sequences, and misassemblies in repetitive regions could introduce errors in the IRs detected. To minimize these effects, we prioritized high-quality complete assemblies and incorporated telomere-to-telomere resolved human (Nurk et al. 2022) and non-human primate genomes (Yoo et al. 2025). Additionally, we included recently published plant assemblies, including *Solanum tuberosum*, *Ziziphus jujuba*, *Musa acuminata malaccensis*, *Glycine max*, *Citrus × limon,* and *Vitis vinifera*. As more T2T assemblies across diverse taxa become available, sequencing and assembly artifacts will be substantially reduced, enabling more accurate detection of IRs.

We conclude that IRs are highly plastic genomic elements, displaying significant variation in frequency across different taxonomic subgroups and within organisms across functional elements.

## Methods

### Data Retrieval and Parsing

Complete genomes were downloaded from the GenBank and RefSeq databases on 3/22/2024 (O'Leary et al. 2016; Benson et al. 2018) using the Genome Updater bash utility https://github.com/pirovc/genome_updater, with the following command:./genome_updater.sh -d “refseq,genbank” -g “archaea,bacteria,fungi,plant,protozoa,vertebrate_mammalian,vertebrate_other,invertebrate,viral” -l “complete genome” -o “assembly_accessions” -f “genomic.fna.gz,genomic.gff.gz” -t 12 -m.

Assembly accessions corresponding to the same accession ID were deduplicated by prioritizing RefSeq entries over GenBank entries. In addition to the large-scale dataset, we explicitly included several high-quality primate assemblies, namely *Gorilla gorilla*, *Pongo pygmaeus*, *Symphalangus syndactylus*, *Pan troglodytes* from (Yoo et al. 2025) and *Homo sapiens* (GCF_009914755.1). Furthermore, we analyzed telomere-to-telomere (T2T) or near T2T assemblies for several additional species that were recently published, including *Solanum tuberosum* (GCA_014189475.1), *Ziziphus jujuba* (GCF_031755915.1), *Musa acuminata malaccensis* (GCA_030219345.1), *Glycine max* (GCA_030864155.1; GCA_033623075.1), *Citrus × limon* (GCA_034663535.1), and *Vitis vinifera* (GCF_030704535.1). These assemblies provide near-complete or complete chromosomal representations and reduce uncertainties associated with repetitive regions.

This process yielded a total of 118,019 complete organismal genomes across the three domains of life and viruses, which were subsequently analyzed and integrated into the database. Gene annotation files in the form of GFF files were downloaded for each genome from the same source. Coordinates for genes, exons, and CDS regions were derived using BEDTools (Quinlan and Hall 2010) and custom awk, bash, and Python scripts. These coordinates were then examined for IR densities across organismal genomes. TSSs and TESs were derived from the corresponding gene coordinates.

### Identification of IRs in Organismal Genomes

IRs with arm lengths equal to or longer than 10 bps, spacer lengths less than nine bps, and without mismatches in the arms were used throughout the study. For IR detection, a modified version of the non-B gfa package was developed and wrapped in a Python program (Cer et al. 2013). The subprocess call to the C script utilized the parameters *minIRrep = 10* and all necessary skip flags to extract only the IR sequences present in each organismal genome. A custom script was used to manually transform the data into a processable tabular format and extract the corresponding arm and spacer sequences along with their corresponding lengths based on the coordinates of the IR sequence. The detected IRs were then systematically validated.

### Calculation of Average IR Density in Each Taxonomic Rank

Each species *S* is represented as a set of individual NCBI assembly accessions $\left\{A_{1},A_{2},\ldots ,A_{n(S)}\right\}$, where n(S) represents the total number of distinct complete genomes that are of type *S*. For each taxonomic rank *R*, containing a collection of species $\left\{S_{1},S_{2},\ldots ,S_{m(R)}\right\}$, where m(R) denotes the total number of distinct species of rank *R*, we calculate the IR density per kB, denoted as F(R), as the average of IR densities across all the species of type *R*, i.e.:


$$F(R)=\frac{\sum_{\;j=1}^{m(R)}\;f(S_{j})}{m(R)},$$

where $f(S_{j})$ represents the individual IR density per kB on the species levels, defined similarly as the average of IR density across all the assembly accessions.

### Derivation of the Tree of Life

We used the NCBI taxdump database to retrieve the full taxonomic lineage for each genome assembly accession. This was done using the ncbitax2lin Python package, which maps species-level taxonomic identifiers (taxIDs) to their complete lineage, spanning all ranks from family to domain. To ensure comprehensive taxonomic coverage, we also incorporated metadata from the NCBI assembly_summary file, which provides detailed lineage information for each genome in our dataset. Since some groups—such as protists—are not explicitly annotated, we used the *group* column in the assembly summary to assign unclassified entries to higher taxonomic categories. For example, assemblies listed under the “protozoa” group were assigned to the Protista kingdom. Similarly, we used the *group* column to fill in missing lineage information at the kingdom and domain levels; for instance, genomes assigned to the “fungi” group were mapped to the Fungi kingdom and the Eukaryota domain.

### Examination of IR Density Across Taxonomic Subdivisions

Organismal genomes were subdivided into different taxonomic levels, namely the three domains of life and Viruses, as well as kingdoms and phyla. The distribution and number of IRs were examined between taxonomic groups at the same taxonomic level by aggregating organisms within each taxonomic group. The density of IRs was defined as the number of IR bps divided by the total bps in a genome or genomic sub-compartment, multiplied by 1,000 to present the density in IR bps per kB. For each domain, kingdom, and phylum, the average IR density (kB) of the associated organismal genomes was calculated. Error bars were determined using the standard error.

### Examination of Biophysical Properties of Inverted Repeats

The number of IRs identified was calculated separately for each combination of spacer and arm length, and the proportions were compared between different taxonomic groups. The mononucleotide and dinucleotide compositions, as well as the GC and AT content of spacers and arms of IRs, were calculated and examined for different arm and spacer lengths. These comparisons were performed across different taxonomic levels.

Using the Jellyfish program (Marçais and Kingsford 2011), we counted the occurrences of mono- and dinucleotides in IR spacers and arms, as well as across each organismal genome. We then performed the enrichment comparison between the IR frequency and the genome frequency of the different mono- and dinucleotides. Word cloud plots were constructed by including only IRs that appeared exclusively in one of the four domains, in order to provide insights specific to that domain.

### Identification of IRs in Simulated Organismal Genomes

To estimate the expected abundance of IRs, we used the uShuffle package (Jiang et al. 2008). For each organismal genome, we employed the Shuffle class from the uShuffle Python package to generate a dinucleotide-preserving shuffled version of the genome. Genomes were processed in non-overlapping chunks of 1,000 base pairs, with each segment shuffled independently to preserve its local dinucleotide structure. The resulting shuffled genome thus consisted of multiple such shuffled segments. This procedure was applied to all available chromosomes of each genome, producing a dinucleotide-frequency-preserving control genome for each organismal genome.

IRs were then extracted from the simulated genomes using the same method as for the original sequences. We compared IR densities (per kilobase) between the shuffled and original genomes, quantifying enrichment as the ratio of observed to expected IR base pairs using the following formula *FE*(*O*,*E*;*G*) = (*O*−*E*)/(*O*+*E*), i.e. (Observed − Expected)/(Observed + Expected), when O≠E and FE(O,E;G)=0, when O=E, where FE(O,E;G) is the enrichment, *O* is the observed, and *E* is the expected IR base pairs in a given organismal genome *G*. Given the definition above, the enrichment function FE(O,E;G) takes values in the interval [−1,1], with the extreme values −1 indicating IR depletion and 1 maximal enrichment, respectively.

To assess the statistical significance of enrichment between organismal genomes and their corresponding dinucleotide-preserving shuffled controls within each taxonomic rank, we compared the distributions of observed and expected IR densities using a two-sided Wilcoxon signed-rank test, with *P*-values adjusted for multiple testing using the Benjamini–Hochberg procedure.

To estimate the magnitude of group differences, we employed two effect size measures: Hedges’ *g* and Cliff's *δ*. Let *N* denote the number of organismal genomes, and let $\left(X_{i},Y_{i}\right)$ represent the paired observed and expected IR densities between the real and the simulated genomes for each 1≤i≤N, respectively. We define the paired differences as *di* = *Xi*$-Y_{i}$. Hedges’ *g* was calculated as follows:


$$g=J(N-1)\frac{\underline{d}}{s_{d}},$$

where $\underline{d}$ and $s_{d}$ denote the empirical mean and standard deviation of differences $d_{i}$, respectively, and J(N−1) is defined as follows:


$$J(n)=1-\frac{3}{4n-1}.$$

Cliff's *δ* was defined for paired differences as follows:


$$\delta =2P(X_{i}>Y_{i})+P(X_{i}=Y_{i})-1,$$

where $P(X_{i}>Y_{i})$ denotes the probability that the observed IR density exceeds that of the matched shuffled genome, and $P(X_{i}=Y_{i})$ denotes the probability of equal densities.

### Progressive Alignments in *Enterococcus faecium*

We downloaded the three *E. faecium* assemblies GCF_900635415.1, GCF_900639415.1, and GCF_900639715.1, directly from NCBI RefSeq database using NCBI datasets CLI tool. We performed pairwise whole sequence alignments in the selected plasmids that large IRs were detected: NZ_LR134107.1 (GCF_900635415.1), NZ_LR135245.1 (GCF_900639415.1), and NZ_LR135490.1 (GCF_900639715.1). The alignments in selected plasmids were performed using minimap2 (Li 2018). The progressive alignment visualizations were performed in Rstudio using SVByEye R program (Porubsky et al. 2025).

### Inverted Repeat Topography Across Functional Genomic Sub-compartments

For each organismal genome, the density of IRs in intergenic, genic, exonic, 5′ UTR, 3′ UTR, and CDS regions was calculated using publicly available GFF coordinate files. A workflow program was developed to process and handle subtle inconsistencies in the downloaded GFF files. To estimate the total IR coverage, missing transcripts and exonic regions of protein-coding genes from prokaryotic assemblies were filled in for each GFF file. Since 3′ and 5′ UTR regions are not present by default, a custom script was used to derive these regions. Subsequently, for each annotated subcompartment, overlapping compartments of the same type were merged and expanded into non-overlapping, mutually disjoint sets of coordinates. These coordinates were then used to estimate the total coverage per Mb in each organismal genome by calculating the total number of bps of IRs divided by the total compartment length of the previously merged genomic regions. Finally, all aggregated statistics for each organismal genome were concatenated into a large, snappy-compressed parquet file, which was then processed to generate the depicted figures.

The density of IRs was calculated at each position within a window relative to the TSS and TES. Enrichment was estimated as the number of IR bps at a position divided by the mean number of bp occurrences across the window of the distribution, as previously described (Georgakopoulos-Soares et al. 2022b). Confidence intervals in the distribution plots were calculated as the standard error at each position.

The IR density in genic, exonic, and CDS regions was calculated by dividing the raw total number of IR overlap bps by the total compartment length in bps and then multiplying the resulting density by 1,000 (kB).

The enrichment density plots across the tree of life were generated by expanding the window size by 500 bps upstream and downstream from the TSS and TES. For each organismal genome, an interval of 1001 bps was constructed and summed symmetrically to determine the total number of IR bps at each coordinate, with 0 representing either the TSS or TES. Each genomic interval was divided by the window mean to account for differences in genome size, as previously described (Georgakopoulos-Soares et al. 2022b). Finally, for every domain, each genomic location was averaged across all organismal genomes to generate the enrichment density plots.

### Term-seq Analysis

We used Term-seq data for *Bacillus subtilis strain 168* from (Kosiński et al. 2025). Subsequently, we calculated the total occurrences of IRs relative to the POT, expanded in a symmetric window of 100 base pairs upstream and downstream.

### Intraspecific Polymorphisms Within IR Loci

The intraspecific variants were obtained from (Chantzi and Georgakopoulos-Soares 2025). We retained only high quality variants with at least 50 minimum mapping quality and 60 Phred score. This was achieved using bcftools filter command. Each site was treated as a binary indicator of an SNV event. The baseline SNV density *r* was defined as the total number of SNV sites per genome, normalized by genome length and computed independently of allele frequency. For a given species, the expected number of SNV sites, based on the total length and the underlying baseline substitution density, was given by the formula E(L,r)=Lr, where *L* is the total genome-wide number of non-overlapping IR base pairs and *r* the genome-wide substitution site density in a given genome *G*. The expected substitution burden based on trinucleotide content adjustment was calculated as follows: Let $p_{j}$ denote the substitution genome-wide frequencies of the 32 canonical trinucleotides (here, canonical stands for minimal to the usual lexicographic order). Let $c_{j}$ be the occurrences of trinucleotide *j* within IR loci. Then, the expected substitution burden is calculated as the expected value of a substitution $E[X]=\overset{32}{\underset{\;j=1}{\sum }}\;c_{j}p_{j},$ where $X=\overset{32}{\underset{\;j=1}{\sum }}\;X_{j}$ is the random variable of the total substitution burden, and $X_{j}$ denotes the random variable of the total substitution burden in the trinucleotide context *j* across $c_{j}$ genomic loci.

For each genome, we compared the expected and observed substitution burden in IR arms and spacers, separately. The effect sizes were calculated as described in the methods section **Identification of IRs in simulated organismal genomes.** Additionally, the deviation of the regression lines from the expected 45 degree line of the first quadrant was quantified as follows:


$$R=\frac{\left|arctan(\beta )\;-\frac{\pi }{4}\right|}{\left|arctan(\alpha )\;\;-\frac{\pi }{4}\right|},$$

where *β* and *α* denote the slopes of the regression lines of IR arm and spacers against expectation, respectively.

### Statistical Analyses

Statistical analyses were performed in Python using the libraries Scipy (Virtanen et al. 2020), and NumPy (Harris et al. 2020).

## Supplementary Material

evag089_Supplementary_Data

## Data Availability

All the source data and the scripts in this study can be found in Zenodo repository (https://zenodo.org/records/18463279). All code is released under the MIT License.

## Code Availability

The code to perform these analyses can be found at: https://github.com/Georgakopoulos-Soares-lab/tree_of_life_IR.
