# Supplementary material for: Characterization of Hairpin Loops and Cruciforms Across 118,019 Genomes Spanning the Tree of Life
Source: Genome Biol Evol. 2026 Apr 4;18(5):evag089. doi: 10.1093/gbe/evag089 (PMC13155455; doi:10.1093/gbe/evag089)
Supplement: evag089_Supplementary_Data [file evag089_supplementary_data.docx]

**Supplementary Figures**


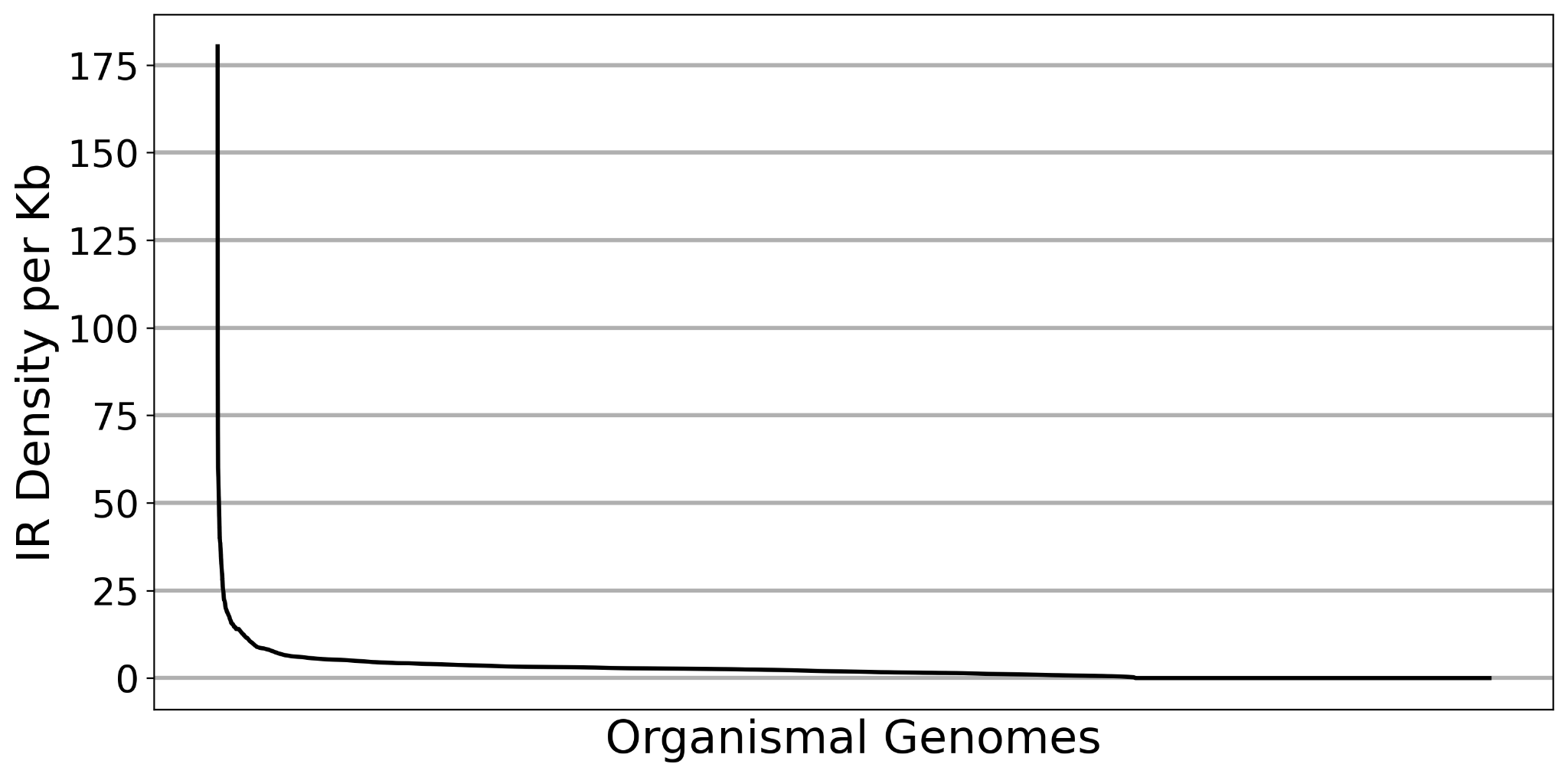


**Supplementary Figure 1: Distribution of the IR density per kB across the organismal genomes studied.**


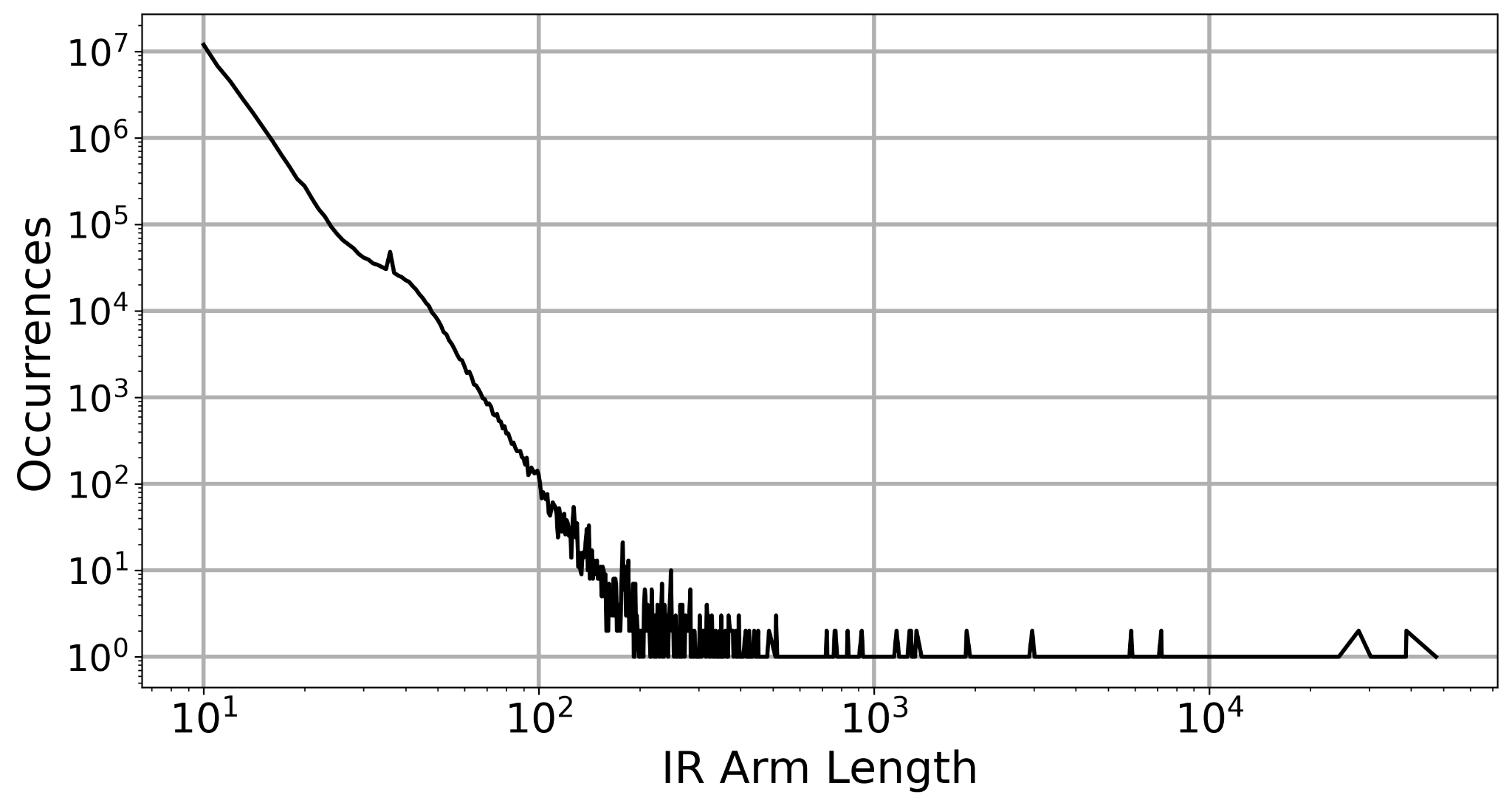


**Supplementary Figure 2: Number of IRs found for different arm lengths.**

**
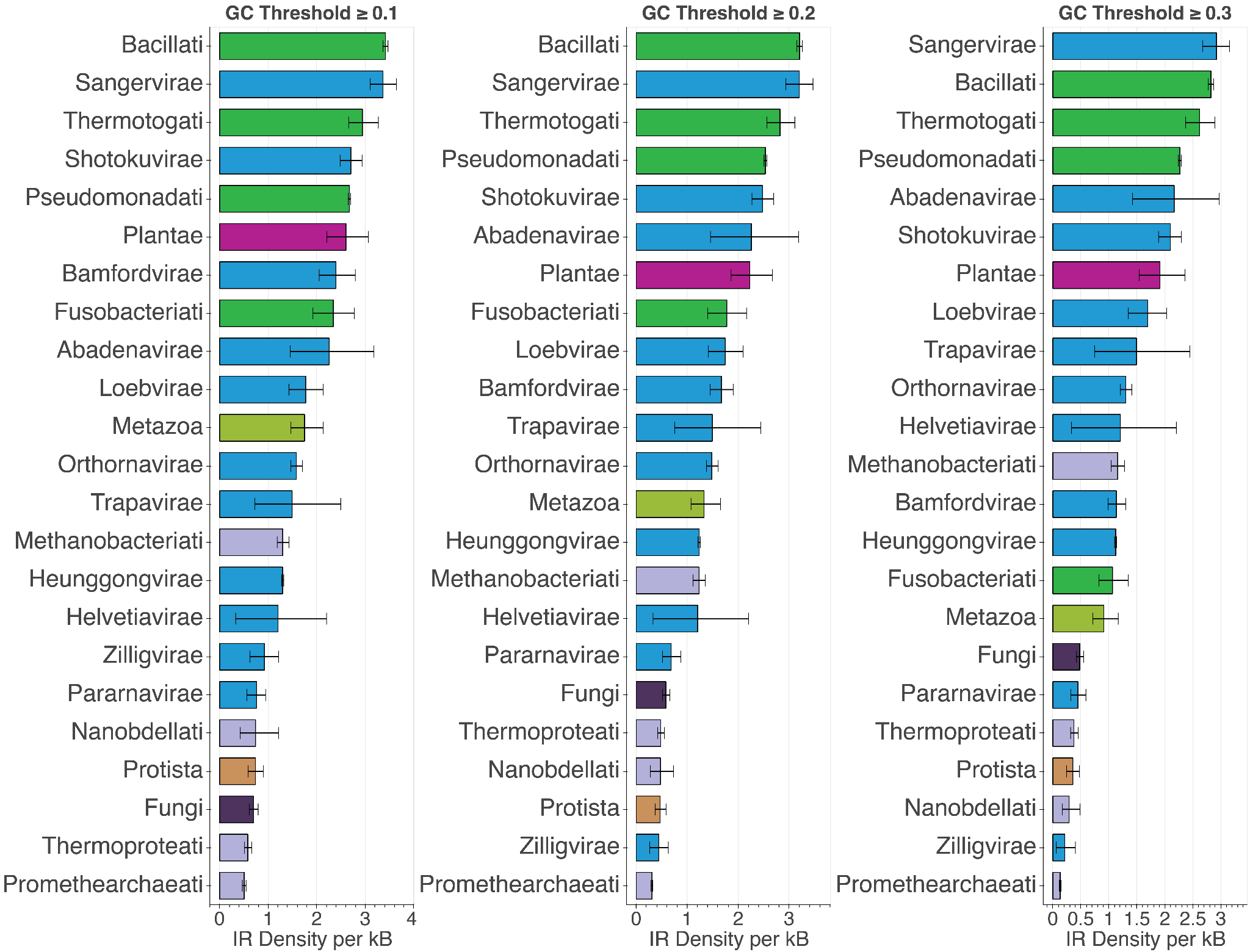
**

**Supplementary Figure 3: IR density across species in the different kingdoms for different thresholds of the GC content of the IR arm sequence.** Results shown for GC% higher than or equal to: a. 10%, b. 20%, c. 30%.


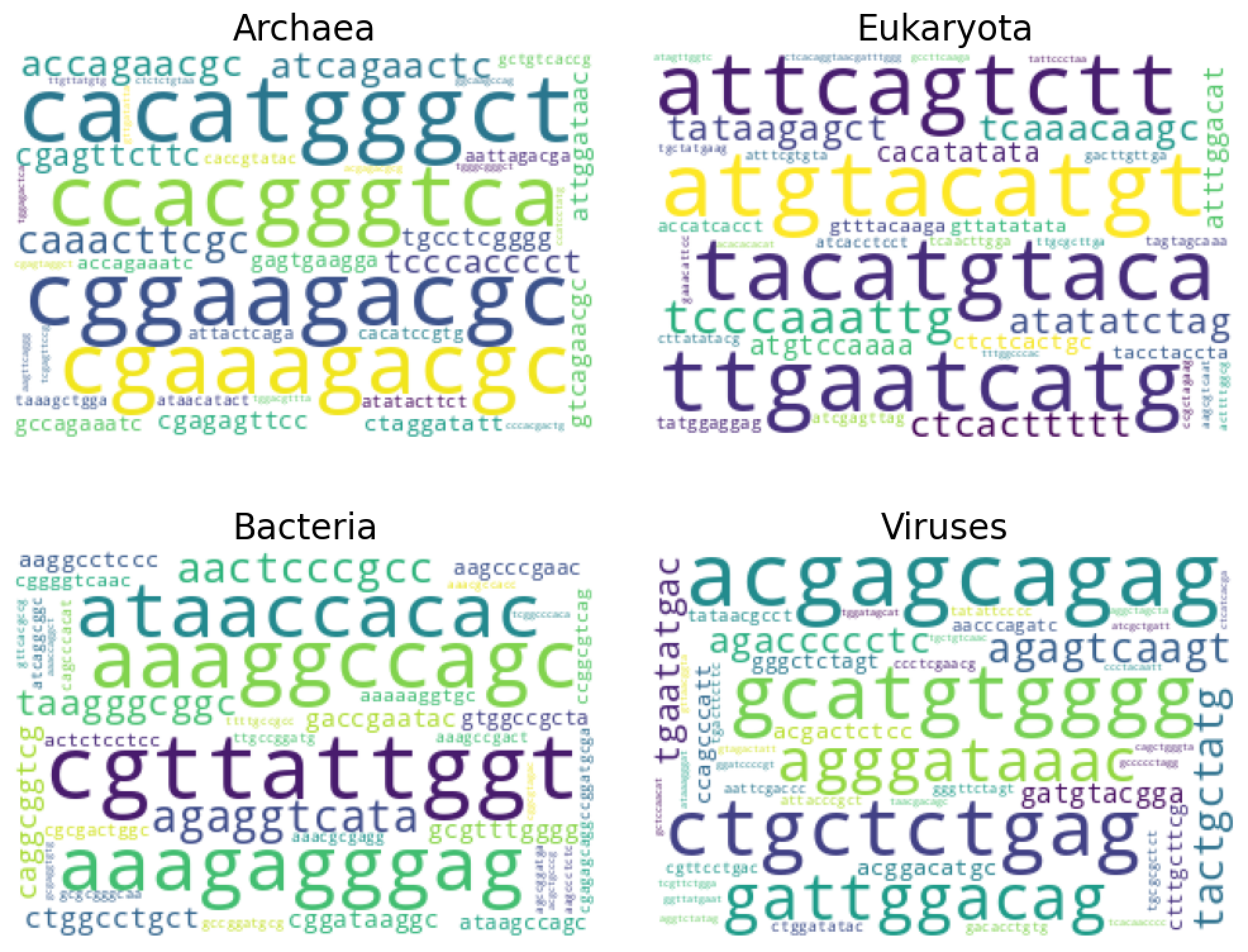


**Supplementary Figure 4: Unique IR arms in each of the three domains of life and Viruses.** Results shown for arm length of ten bps. The font size represents the frequency of each IR arm sequence.

**
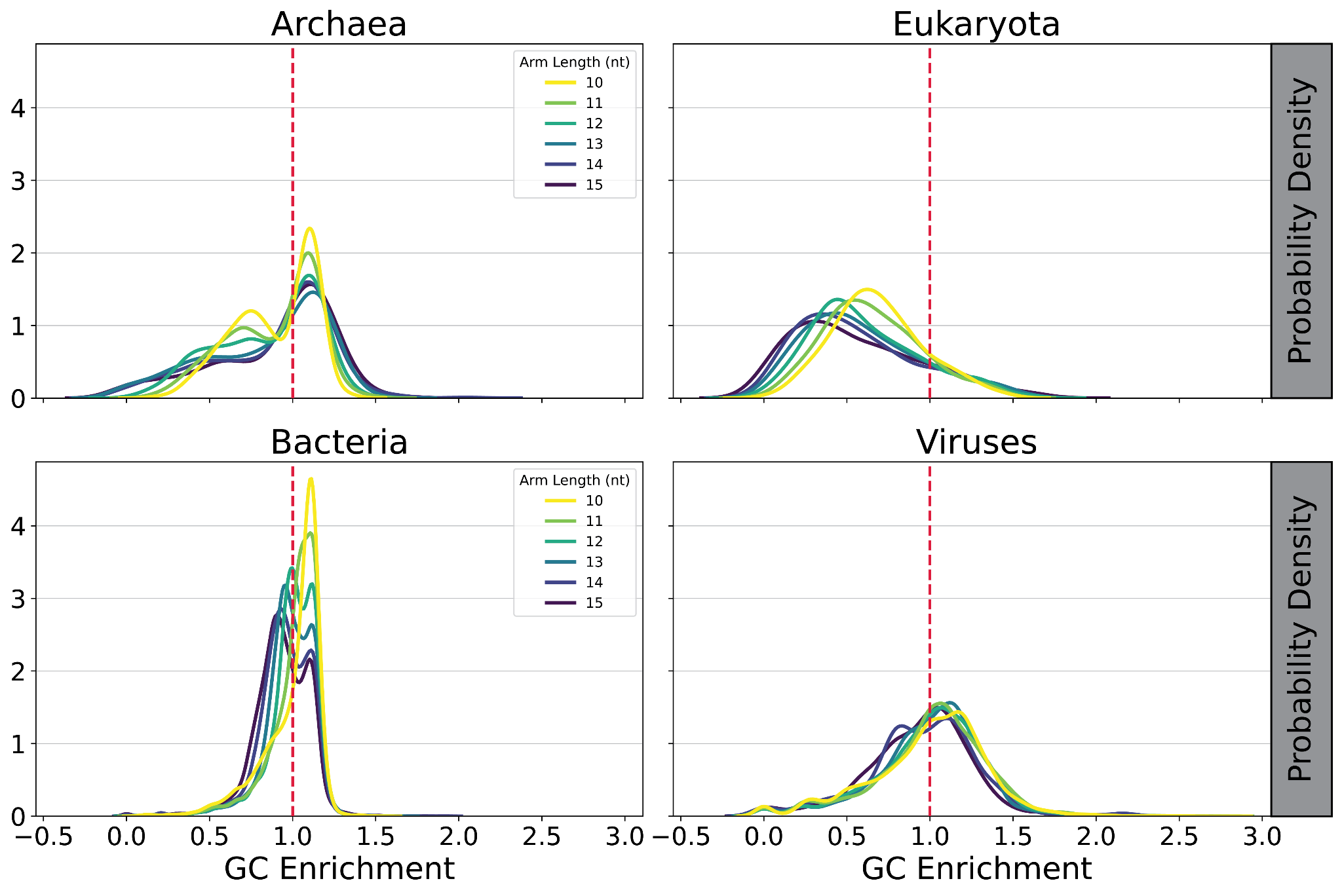
**

**Supplementary Figure 5: Kernel density estimation of IR arm GC enrichment relative to the genome-wide background rate across species spanning the three domains of life and Viruses partitioned by the IR arm length.**


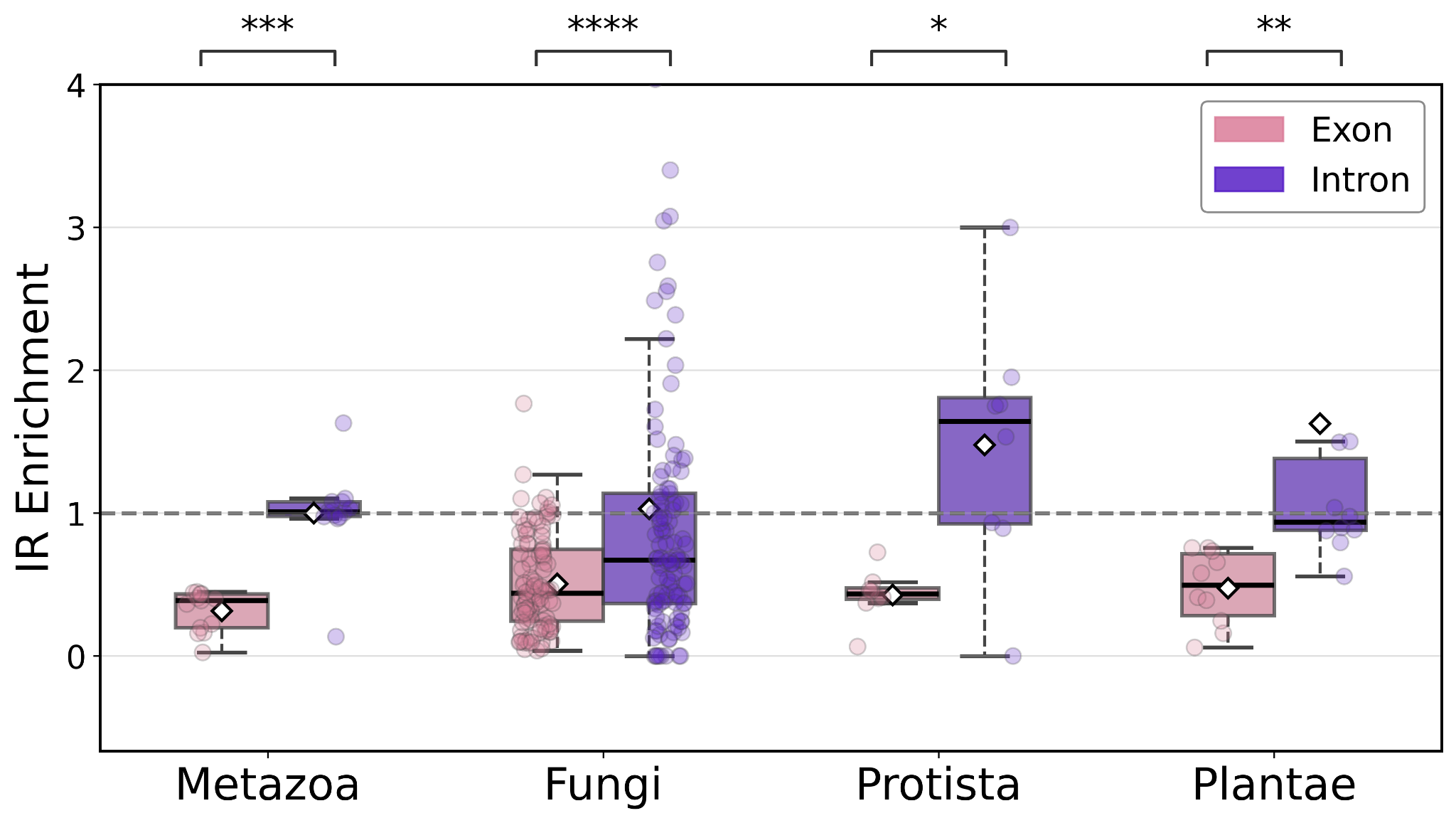


**Supplementary Figure 6: Comparison of normalized IR density in intronic and exonic regions across eukaryotic kingdoms.** Statistical significance was assessed using a two-sided Wilcoxon signed-rank test, and p-values were adjusted for multiple hypothesis testing using the Benjamini–Hochberg procedure. Adjusted p-values are displayed as * for p < 0.05, ** for p < 0.01, *** for p < 0.001, and **** for p < 0.0001.

**
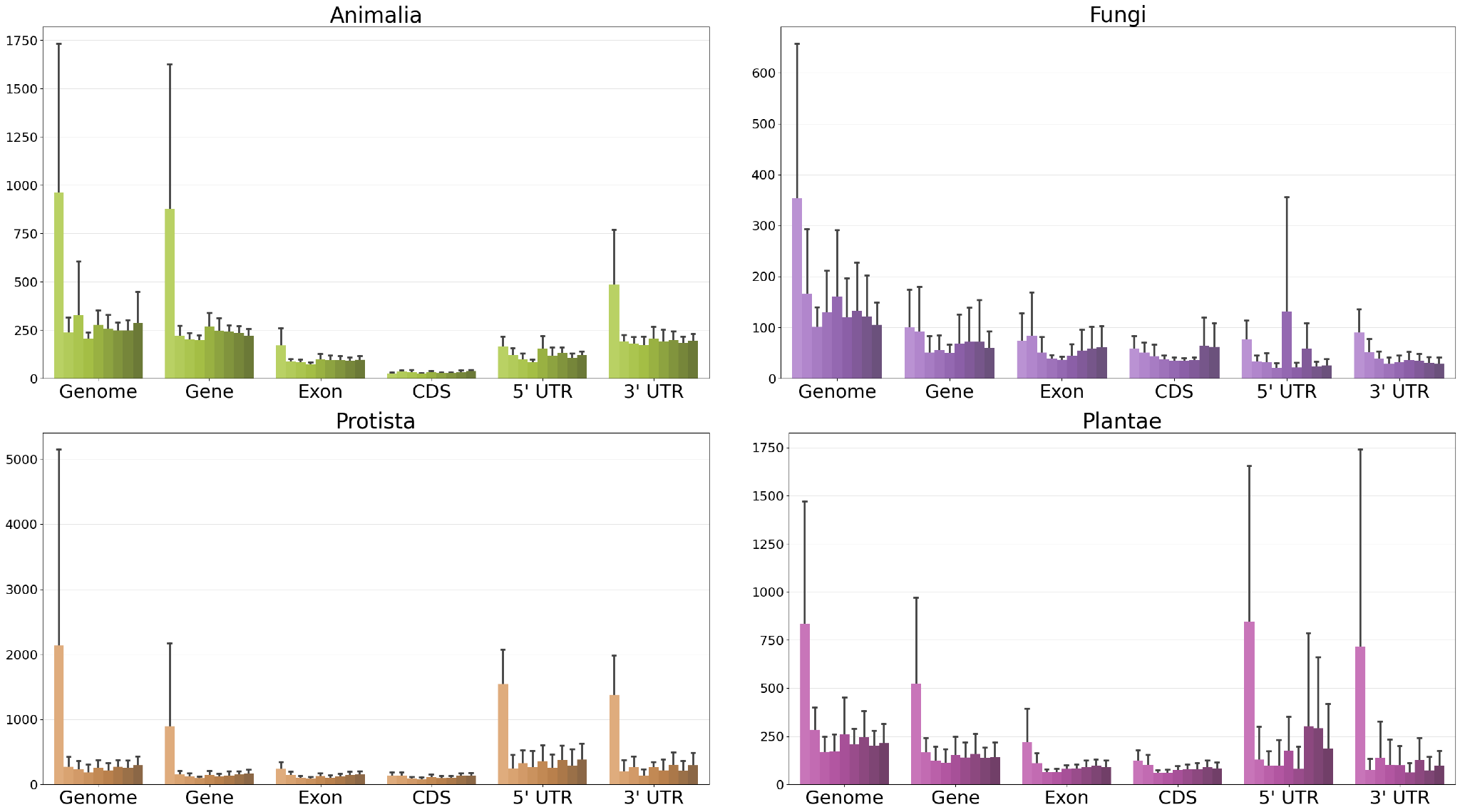
**

**Supplementary Figure 7: Comparison of IR density within eukaryotic kingdoms partitioned by spacer length.**

**
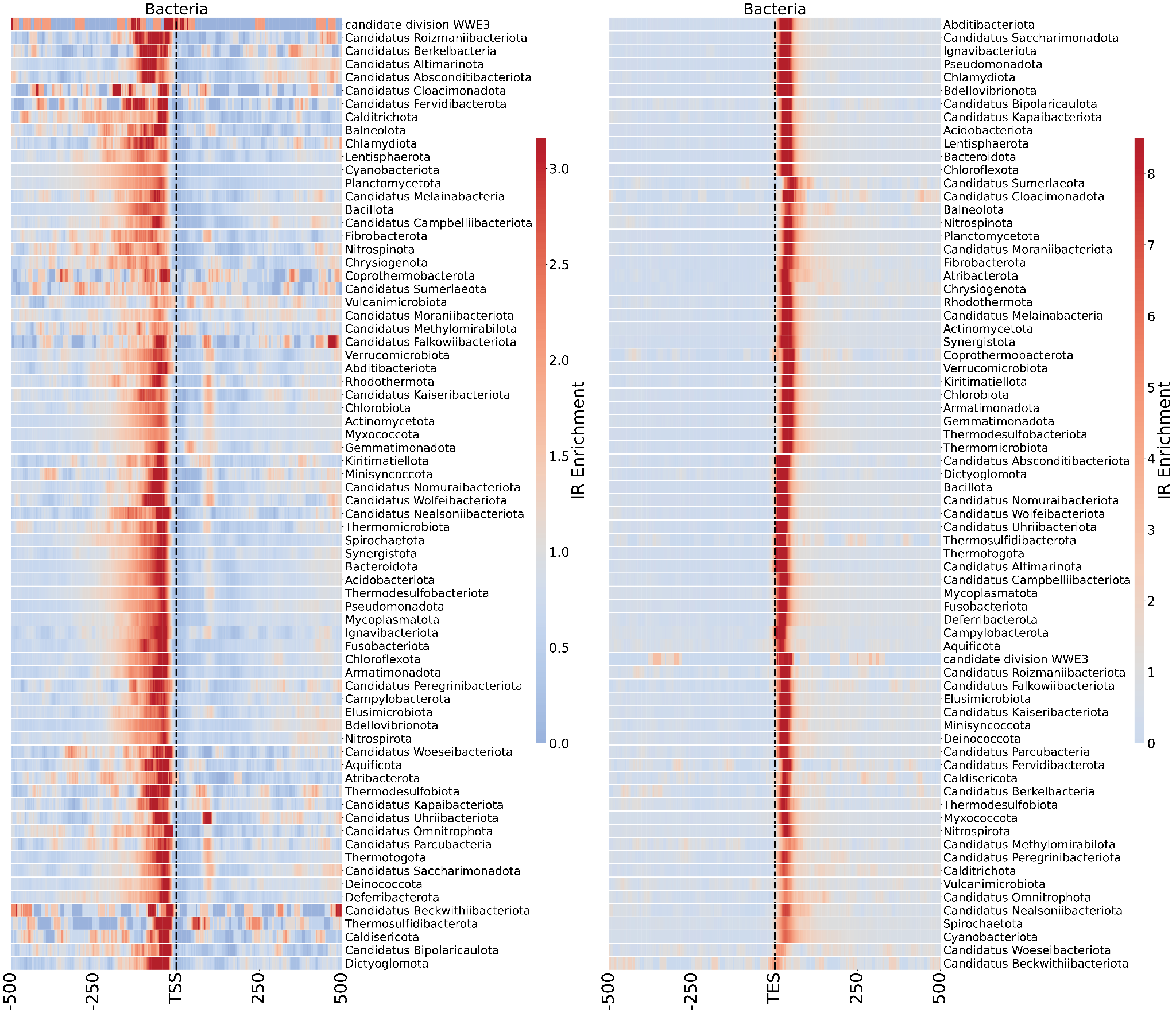
**

**Supplementary Figure 8: Enrichment of IRs relative to the TSS and TES in bacterial phyla.**

**
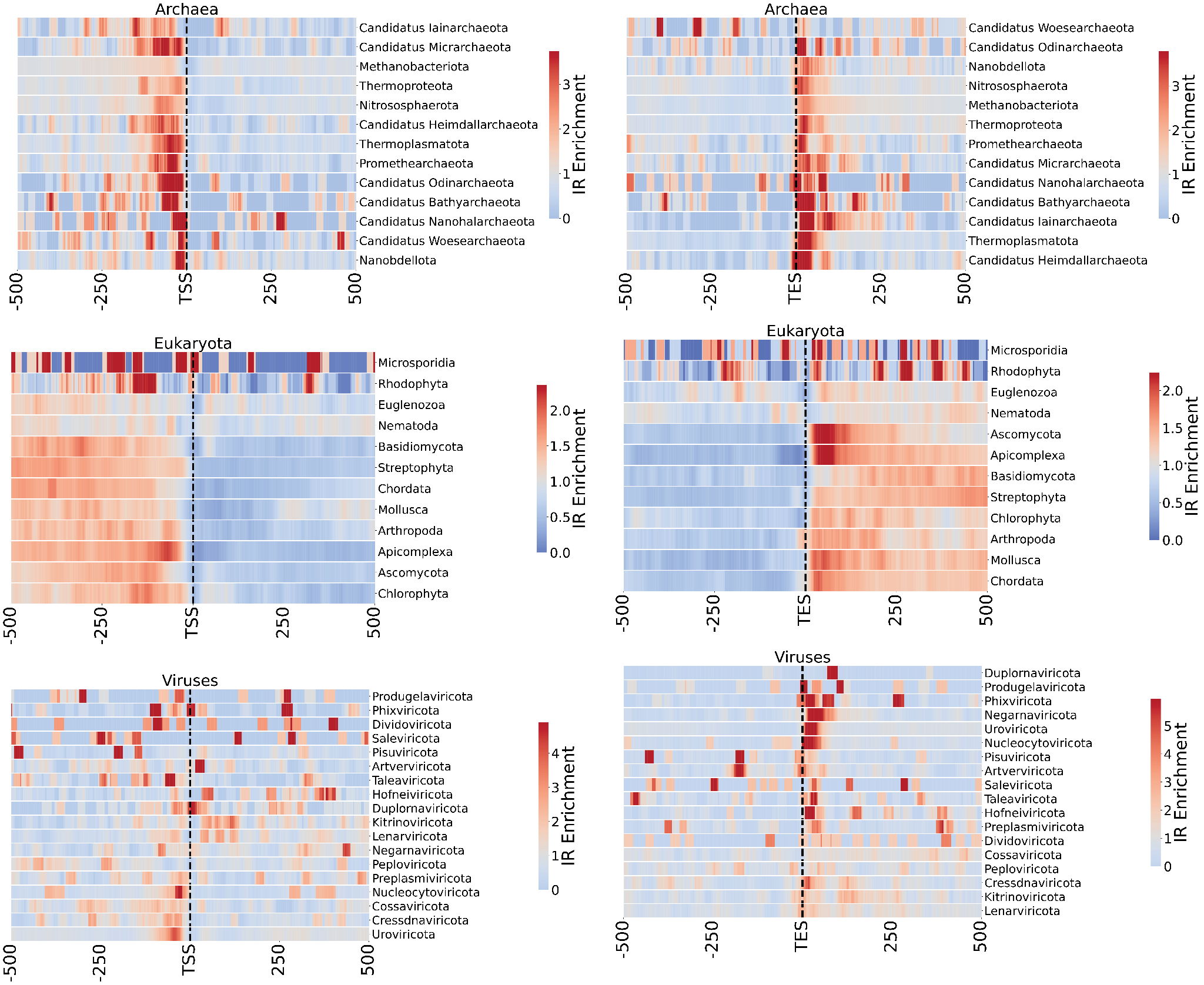
**

**Supplementary Figure 9: Enrichment of IRs relative to the TSS and TES in archaeal, eukaryotic, and viral phyla.**

**
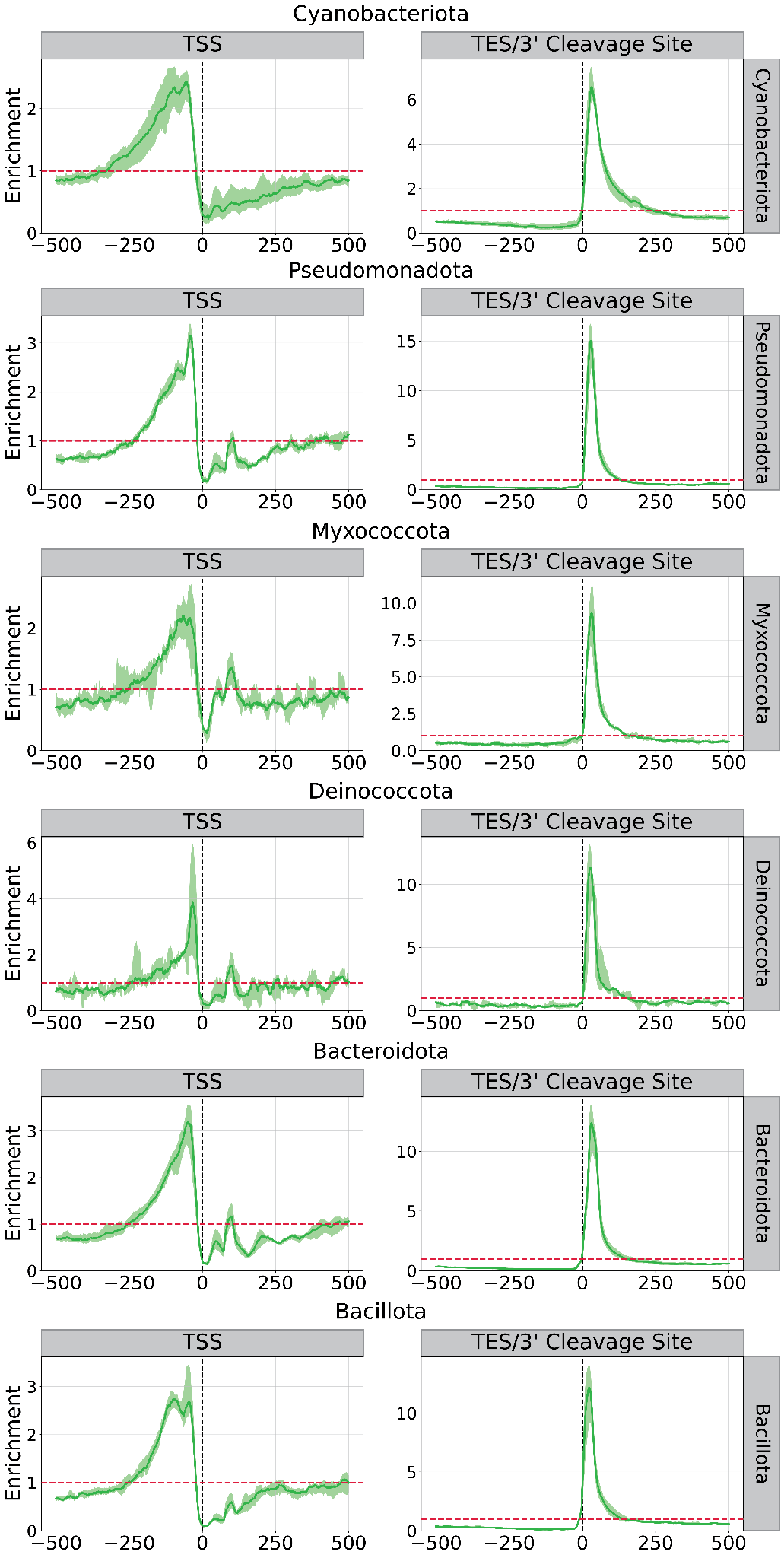
**

**Supplementary Figure 10: Enrichment of IRs relative to the TSS and TES/3' Cleavage Site in individual bacterial phyla.**

**
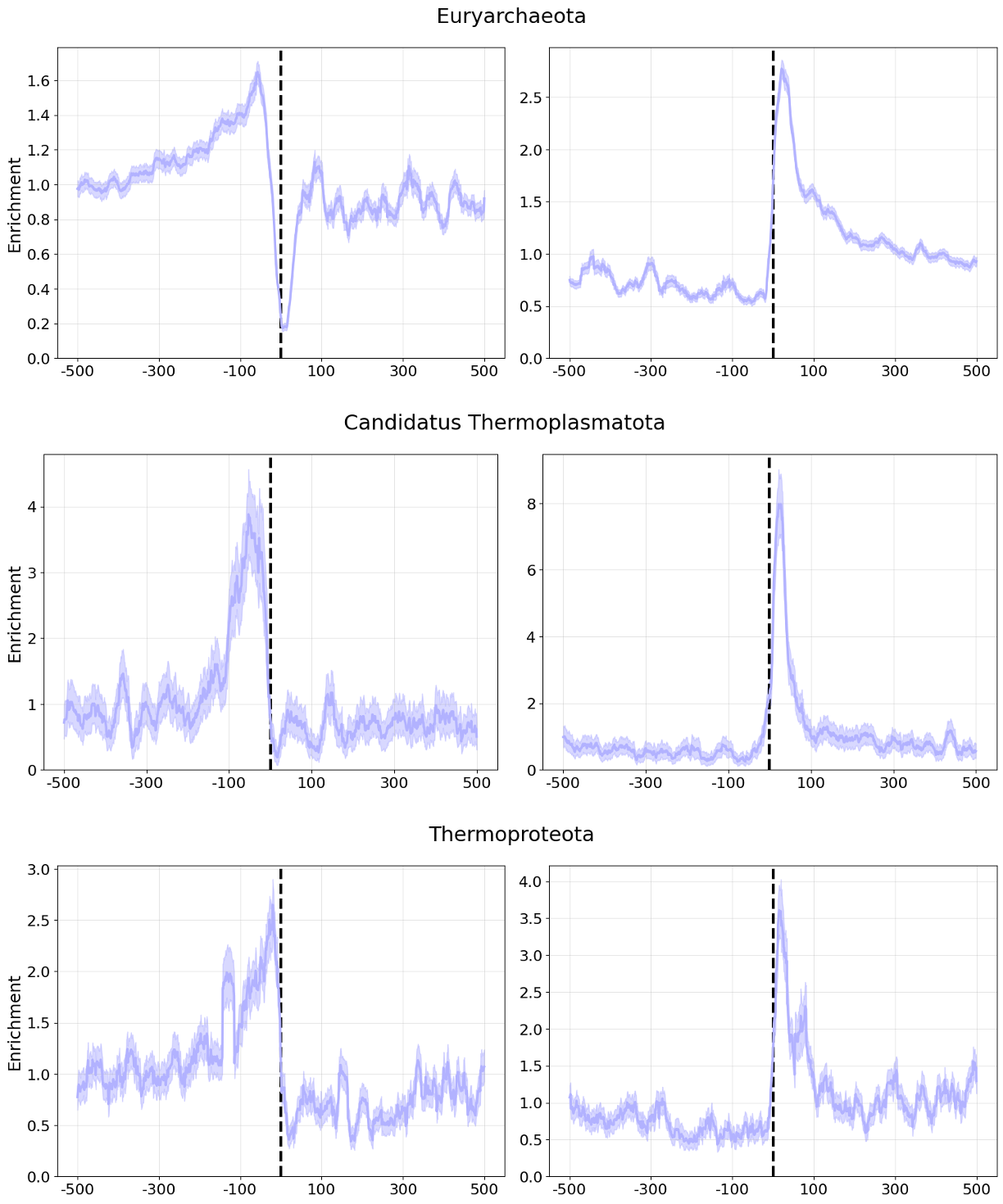
**

**Supplementary Figure 11: Enrichment of IRs relative to the TSS and TES in individual archaeal phyla.**

**
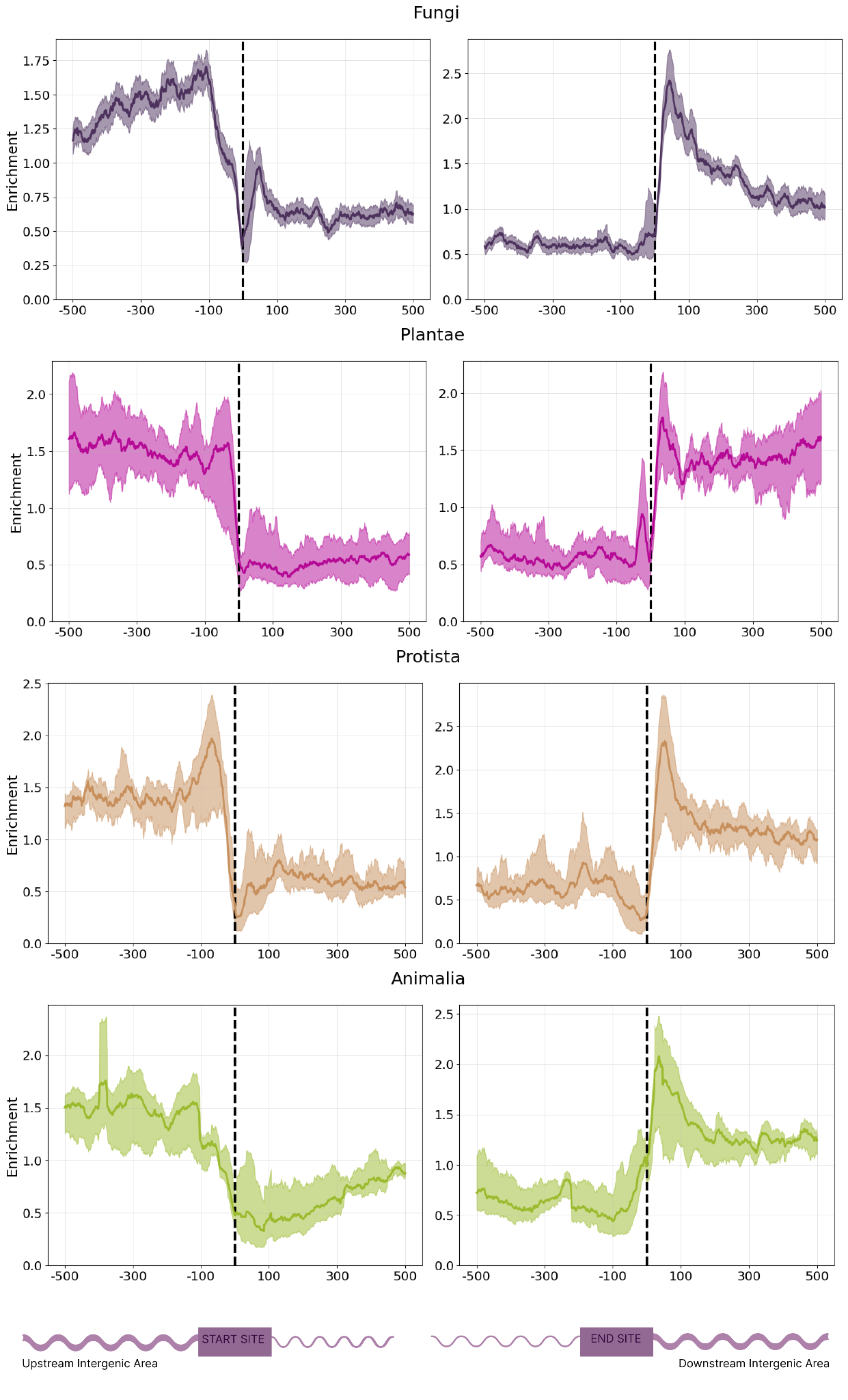
**

**Supplementary Figure 12: Enrichment of IRs relative to the TSS and TES in individual eukaryotic kingdoms.**

**
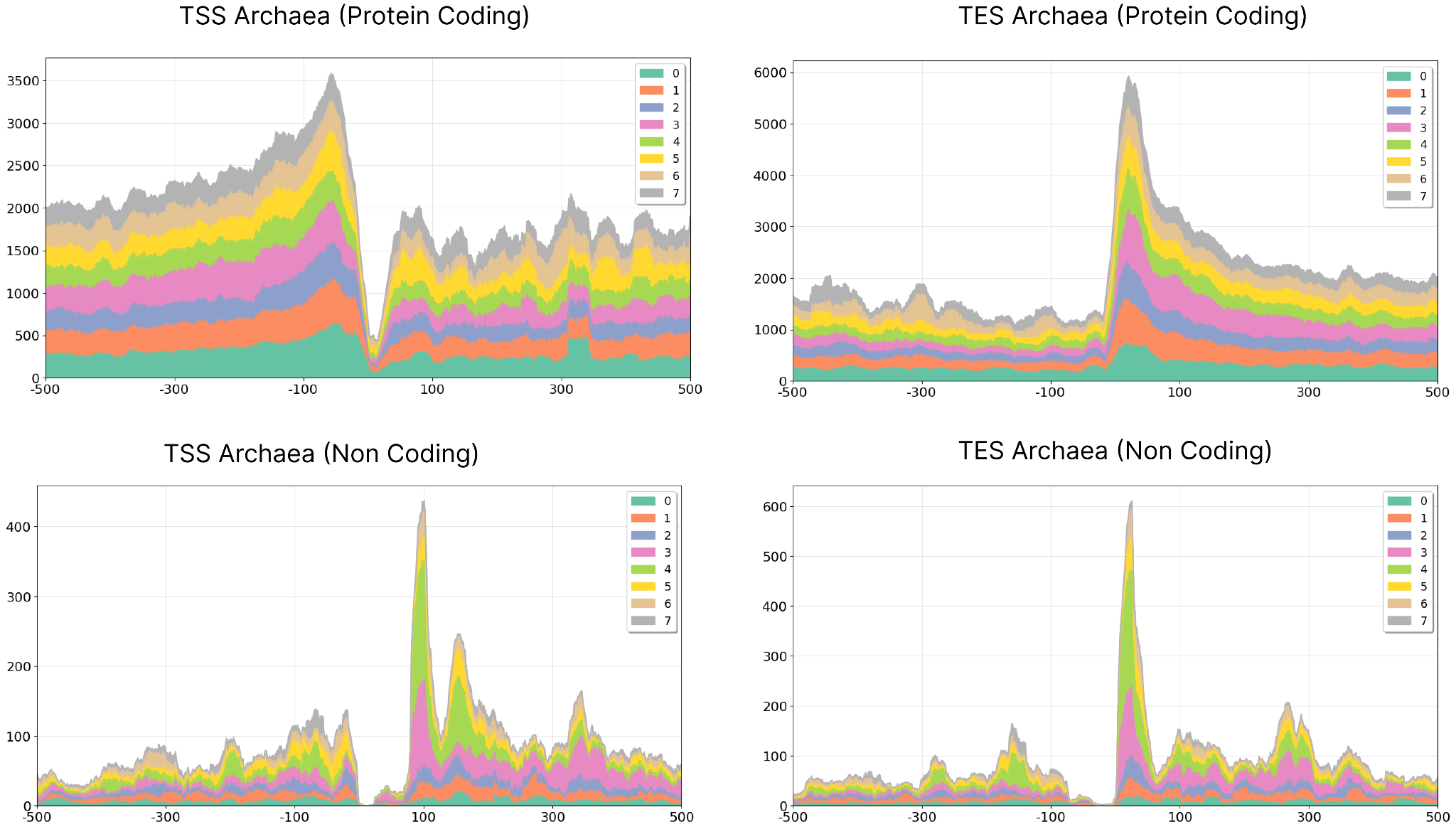
**

**
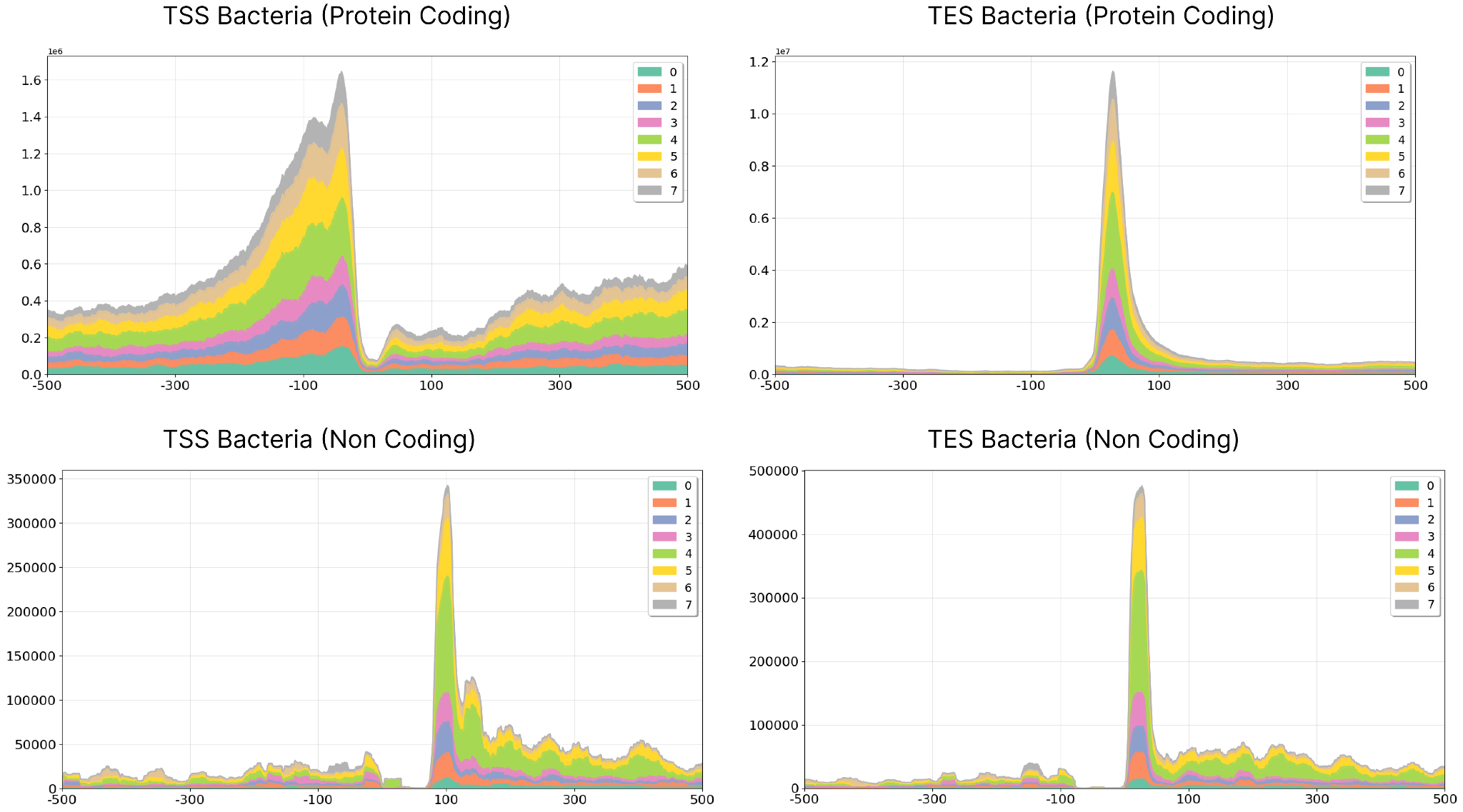
**

**
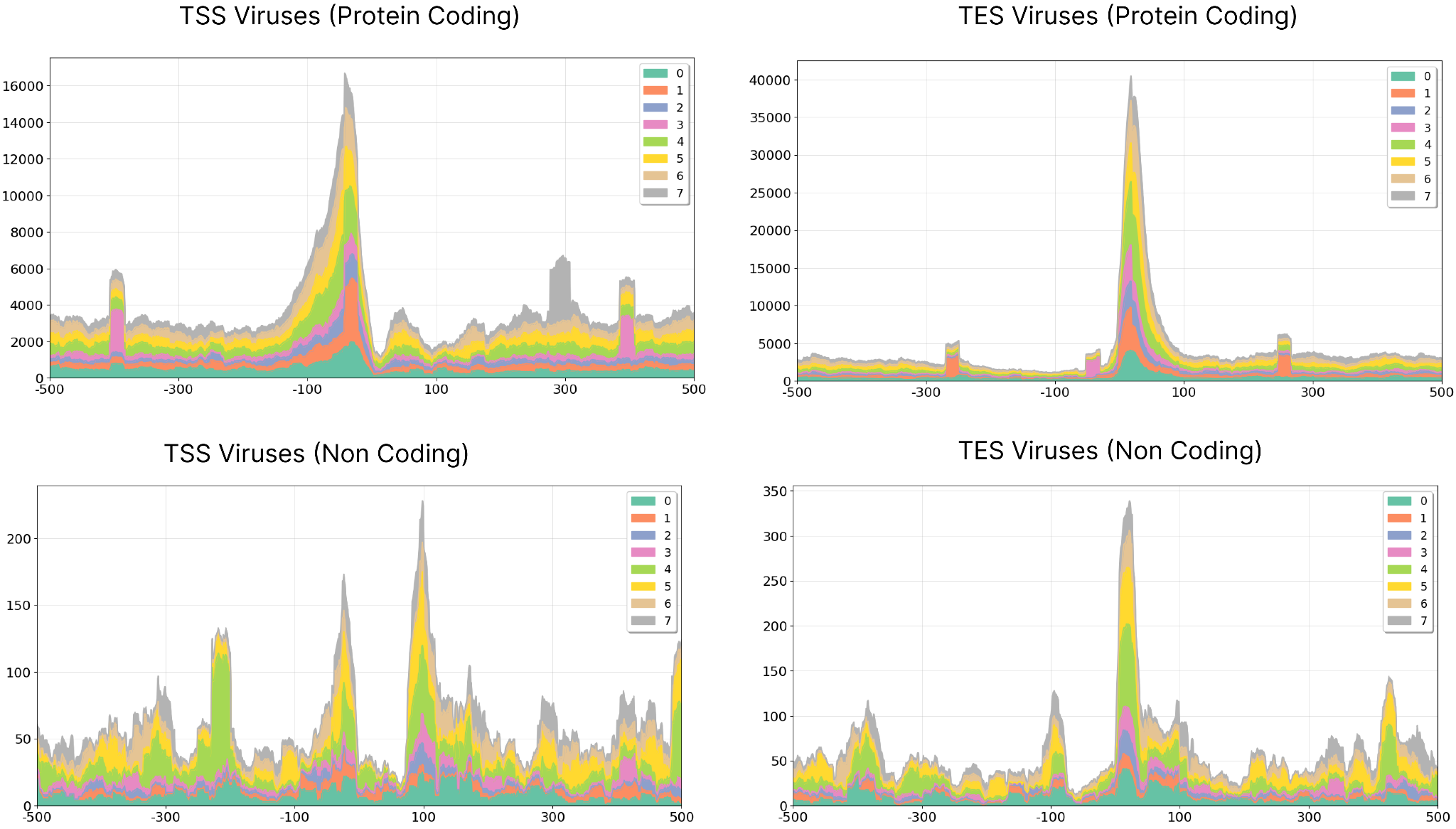
**

**
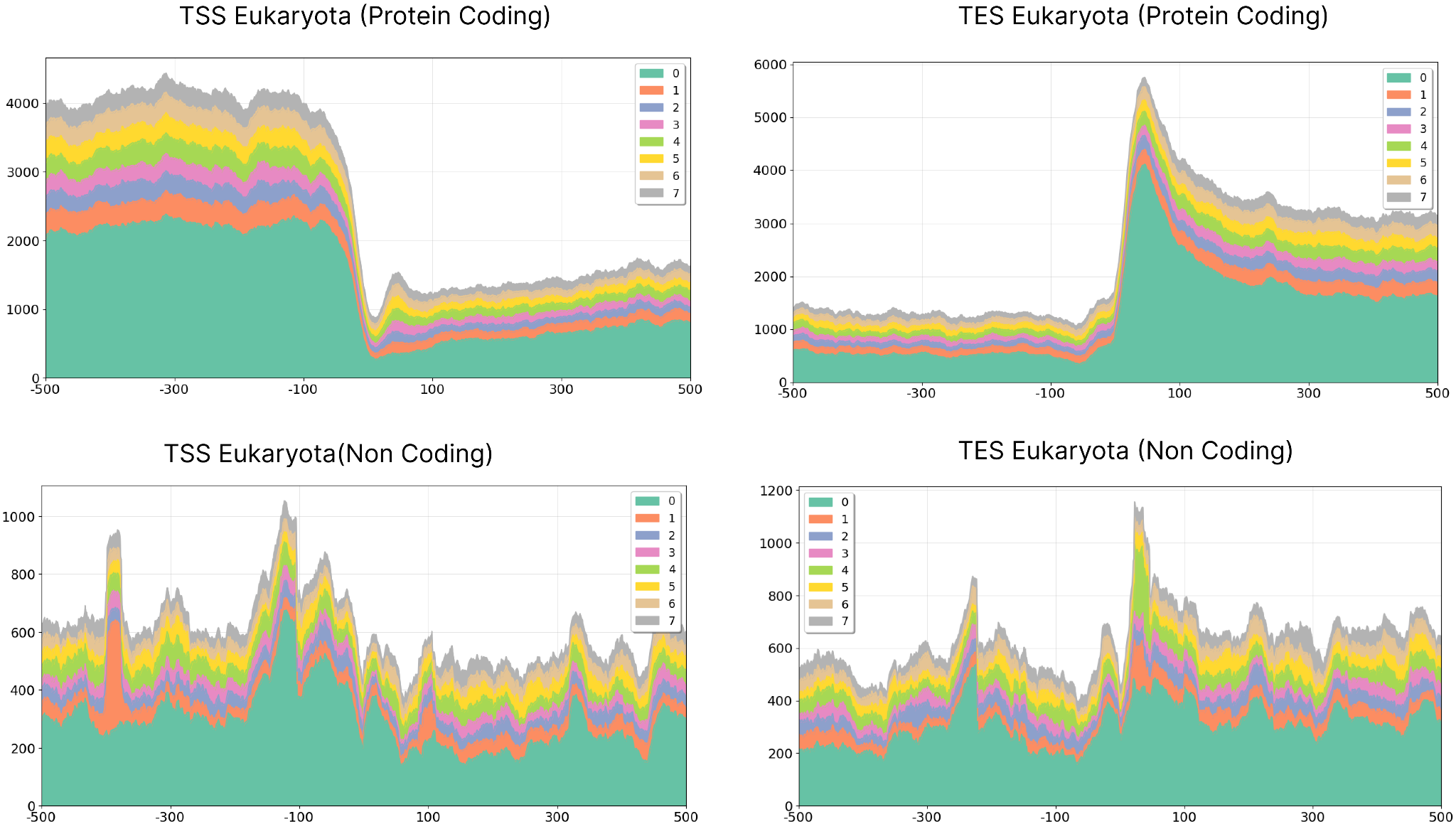
**

**Supplementary Figure 13: Distribution of IRs separated by spacer length, in coding- and non-coding genes.**

**Supplementary Table 1: Database breakdown into the three domains of life and viruses.**

| **Domain** | **Total Genomes** | **Total Unique Species** |
| --- | --- | --- |
| Bacteria | 49,191 | 11,118 |
| Viruses | 67,654 | 32,106 |
| Archaea | 687 | 528 |
| Eukaryota | 487 | 290 |

**Supplementary Table 2: Database breakdown in kingdoms.**

| **Kingdom** | **Total Genomes** | **Total Unique Species** | **Total Phylums** |
| --- | --- | --- | --- |
| Nanobdellati | 15 | 14 | 6 |
| Metazoa | 25 | 22 | 4 |
| Fungi | 381 | 212 | 5 |
| Zilligvirae | 36 | 30 | 1 |
| Pararnavirae | 984 | 267 | 1 |
| Pseudomonadati | 32,296 | 6,528 | 42 |
| Abadenavirae | 25 | 24 | 1 |
| Helvetiavirae | 10 | 9 | 1 |
| Heunggongvirae | 23,300 | 16,725 | 2 |
| Thermoproteati | 164 | 108 | 3 |
| Bacillati | 16,337 | 4,316 | 8 |
| Plantae | 49 | 35 | 3 |
| Shotokuvirae | 8,845 | 2,593 | 2 |
| Thermotogati | 156 | 96 | 3 |
| Bamfordvirae | 6,671 | 410 | 3 |
| Orthornavirae | 14,906 | 6,807 | 7 |
| Trapavirae | 21 | 21 | 1 |
| Loebvirae | 387 | 238 | 1 |
| Sangervirae | 4,382 | 1,033 | 1 |
| Protista | 32 | 21 | 4 |
| Methanobacteriati | 495 | 394 | 2 |
| Fusobacteriati | 124 | 36 | 1 |
| Promethearchaeati | 5 | 5 | 3 |

**Supplementary Table 3: Longest, perfect IRs across the genomes studied.**

| Inverted Repeat Arm Length (bps) | Species | Spacer Length (bps) | Arm GC Content (%) | Sequence ID | Assembly accession (GenBank & RefSeq) |
| --- | --- | --- | --- | --- | --- |
| 47,461 | *Enterococcus faecium* | *0* | *32.814* | NZ_LR135490.1 | GCF_900639715.1 |
| 38,727 | *Olsenella timonensis* | *0* | *69.365* | NZ_LT635455.1 | GCF_900119915.1 |
| 38,727 | *Atopobiaceae bacterium* | *0* | *69.365* | LR698995.1 | GCA_902386885.1 |
| 38,611 | *Rhodococcus sp. AH-ZY2* | *1* | *64.945* | NZ_CP126460.1 | GCF_030916465.1 |
| 38,528 | *Pseudomonas aeruginosa* | *0* | *66.824* | NZ_CP096940.1 | GCF_030121995.1 |
| 37,277 | *Pseudomonas corrugata* | *0* | *60.627* | NZ_CP102178.1 | GCF_026016525.1 |
| 30,325 | *Klebsiella pneumoniae* | *0* | *51.023* | NZ_CP097676.1 | GCF_023573745.1 |
| 27,900 | *Enterococcus faecium* | *0* | *33.225* | NZ_LR135245.1 | GCF_900639415.1 |
| 22,733 | *Staphylococcus pasteuri* | *1* | *30.625* | NZ_CP031281.1 | GCF_007814825.1 |
| 18,812 | *Ligilactobacillus murinus* | *0* | *35.461* | NZ_CP040853.1 | GCF_010586905.1 |
| 18,297 | *Escherichia coli* | *0* | *45.335* | CP088379.1 | GCA_021130595.1 |
| 17,045 | *Acanthamoeba polyphaga mimivirus* | *0* | *26.617* | KM982401.1 | GCA_002966385.1 |
| 14,452 | *Agrobacterium pusense* | *0* | *55.272* | NZ_CP097632.1 | GCF_030440405.1 |
| 13,509 | *Melissococcus plutonius* | *0* | *29.350* | NZ_AP018525.1 | GCF_004001205.1 |
| 13,040 | *Salmonella enterica* | *4* | *49.095* | CP082621.1 | GCA_004280135.2 |

**Supplementary Table 4: Largest perfect IRs detected in *Homo Sapiens*.**

| Chromosome | Start (bp) | End (bp) | Arm Length (bp) | Spacer Length (bp) | GC Content (%) |
| --- | --- | --- | --- | --- | --- |
| chr8 | 125,615,789 | 125,616,077 | 140 | 8 | 2.08 |
| chr8 | 87,939,831 | 87,940,074 | 118 | 7 | 1.23 |
| chr9 | 114,631,600 | 114,631,798 | 95 | 8 | 25.75 |
| chr11 | 42,828,571 | 42,828,757 | 93 | 0 | 18.28 |
| chrX | 138,732,619 | 138,732,799 | 88 | 4 | 37.22 |
| chr3 | 60,903,975 | 60,904,152 | 86 | 5 | 11.86 |
| chr6 | 67,938,601 | 67,938,769 | 84 | 0 | 15.48 |
| chr11 | 5,253,730 | 5,253,890 | 80 | 0 | 37.50 |
| chr2 | 85,010,163 | 85,010,327 | 80 | 4 | 6.71 |
| chr13 | 83,726,139 | 83,726,299 | 80 | 0 | 3.75 |
